# Comparative Efficacy and Safety of P2Y12 Inhibitors in Patients With Diabetes Mellitus and Coronary Artery Disease: A Systematic Review and Network Meta-Analysis of Randomized Controlled Trials

**DOI:** 10.14740/cr2254

**Published:** 2026-07-15

**Authors:** Abdullah Ghazi Aljohani, Osamah Omar Alturki, Ghaida Yahya Alahmari, Abdulrahman Thumayl Al-Shammari, Muqrin Abdullah Alharbi, Asmaa Esam Saeed Ali, Ali Saleh AlYounis, Abdulkarim Abdurrahman Mohammed Alshehri, Fahad Ali Hussain Alqahtani, Nourhan Sulaiman Basyouni, Khalid Noor Almutairi, Refal Hamad Jamjoom, Mansour Mohammed Abdullah Alahmari, Ahmed Sobhy

**Affiliations:** a Department of Internal Medicine, Dr. Sulaiman Al Habib Medical Group, Jeddah, Saudi Arabia; b King Saud bin Abdulaziz University for Health Sciences, Jeddah, Saudi Arabia; c Department of Internal Medicine, Batterjee Medical College, Abha, Saudi Arabia; d Faculty of Medicine, University of Hail, Saudi Arabia; e Faculty of Medicine, Alexandria University, Egypt; f Faculty of Medicine, University of Sana’a, Saudi Arabia; g Department of Clinical Pharmacy, King Faisal General Hospital, Al Ahsa Health Cluster, Al Ahsa, Saudi Arabia; h Department of Internal Medicine, Billasmar General Hospital, Abha, Saudi Arabia; i Medical Services - MOI, Abha, Saudi Arabia; j Department of Internal Medicine, Al Imam Abdulrahman Alfaisal Hospital, Riyadh, Saudi Arabia; k College of Medicine and Surgery, Majmaah University, Riyadh, Saudi Arabia; l Faculty of Medicine, Kafr Elsheikh University, Egypt

**Keywords:** Diabetes mellitus, Coronary artery disease, Prasugrel, Ticagrelor

## Abstract

**Background:**

Patients with diabetes mellitus (DM) and coronary artery disease (CAD) are at high risk of thrombotic events and exhibit reduced responsiveness to conventional antiplatelet therapy. While newer P2Y12 inhibitors such as ticagrelor and prasugrel provide more potent platelet inhibition, the optimal therapeutic strategy in this population remains uncertain.

**Methods:**

A systematic review and network meta-analysis of randomized controlled trials (RCTs) was conducted in accordance with PRISMA guidelines. Electronic databases were searched through April 2026 to identify RCTs comparing clopidogrel, prasugrel, ticagrelor, and placebo in patients with DM and CAD receiving background aspirin therapy. Random-effects models were used to estimate pooled risk ratios (RRs) with 95% confidence intervals (CIs). The primary efficacy outcome was major adverse cardiovascular events (MACEs), while safety outcomes included major bleeding.

**Results:**

Seventeen RCTs, including 40,919 patients, were identified, with 15 trials included in the quantitative synthesis. Compared with placebo, prasugrel and ticagrelor were associated with significant reductions in MACEs, whereas clopidogrel showed no significant benefit. No significant differences were observed in cardiovascular or all-cause mortality across treatment strategies. Prasugrel was associated with a lower risk of myocardial infarction, while ticagrelor was associated with a lower risk of stroke. Both agents increased major bleeding versus placebo, and ticagrelor was also associated with a higher risk of dyspnea. Ranking analysis suggested that prasugrel was the most effective strategy for ischemic outcomes, whereas placebo was the safest strategy overall.

**Conclusions:**

In patients with DM and CAD, prasugrel and ticagrelor provide superior protection against ischemic events compared with clopidogrel but are associated with an increased bleeding risk compared with placebo. Prasugrel appears to offer the greatest overall efficacy, while ticagrelor shows a distinct benefit in stroke prevention. These findings support individualized antiplatelet strategies that balance ischemic and bleeding risks in this high-risk population.

## Introduction

Patients with diabetes mellitus (DM) and concomitant coronary artery disease (CAD) are at increased risk of major adverse cardiovascular events (MACEs), recurrent atherothrombotic complications, and reduced responsiveness to conventional antiplatelet therapies compared with non-diabetic patients [1–4]. These pathophysiological features are driven by a complex prothrombotic and inflammatory milieu, leading to increased platelet reactivity, and underscoring the need for effective antiplatelet therapy [5].

Dual antiplatelet therapy (DAPT), consisting of aspirin in combination with an oral P2Y12 receptor inhibitor, remains the cornerstone of secondary prevention in patients with CAD [6]. Among currently available P2Y12 receptor inhibitors, clopidogrel is the most widely used adjunct to aspirin in patients with CAD and has been shown to reduce ischemic event rates [7]. However, in diabetic patients, clopidogrel has limited efficacy because of altered pharmacokinetic and pharmacodynamic responses, resulting in reduced generation of its active metabolite and higher rates of high on-treatment platelet reactivity, which may contribute to adverse ischemic outcomes [3, 8]. Moreover, prasugrel and ticagrelor are newer-generation P2Y12 inhibitors that provide more rapid, potent, and consistent platelet inhibition than clopidogrel in high-risk populations, including patients with DM. Prasugrel, a third-generation thienopyridine with a more favorable pharmacokinetic and pharmacodynamic profile than clopidogrel [9], whereas ticagrelor, a direct-acting reversible non-thienopyridine P2Y12 receptor antagonist, provides faster onset, more predictable, and more consistent platelet inhibition than clopidogrel [10].

Despite the availability of multiple antiplatelet strategies, the optimal therapy for patients with DM remains uncertain. The comparative efficacy and safety of these treatment options have not been comprehensively evaluated within a single analytical framework. Therefore, network meta-analysis (NMA) provides a robust approach to integrate direct and indirect evidence and to compare and rank antiplatelet therapies according to their effects on ischemic and bleeding outcomes in this high-risk population.

## Materials and Methods

This systematic review and NMA were conducted in line with the Preferred Reporting Items for Systematic Reviews and Meta-Analyses (PRISMA) 2020 statement [11] and followed the methodological guidance described in the Cochrane Handbook for Systematic Reviews of Interventions [12]. The review protocol was prospectively registered in the International Prospective Register of Systematic Reviews (PROSPERO) under Registration Number CRD420261383166. This study is a systematic review and NMA of previously published randomized controlled trials (RCTs), and it did not involve the direct participation of human subjects or access to identifiable patient data.

### Data sources and study selection

We conducted a comprehensive literature search across the following electronic databases: PubMed/MEDLINE, Scopus, Web of Science, and the Cochrane Central Register of Controlled Trials (CENTRAL), from database inception through April 2026. The search strategy was designed to identify RCTs evaluating oral P2Y12 inhibitor–based DAPT in patients with DM and CAD. The core search syntax was as follows: (“coronary artery disease” OR “CAD” OR “acute coronary syndrome” OR “ACS” OR “myocardial infarction” OR “MI” OR “unstable angina” OR “UA”) AND (“diabetes mellitus” OR “DM” OR “diabetes” OR “diabetic”) AND (“clopidogrel” OR “prasugrel” OR “ticagrelor” OR “P2Y12” OR “P2Y12 inhibitor”). In addition, a manual citation search was performed by screening the reference lists of all included studies and relevant review articles to identify any additional eligible studies. Detailed search strategies for each database are provided in the Supplementary Material 1 (cr.elmerpub.com).

All records identified through the literature search were imported into EndNote for deduplication, and the remaining records were subsequently uploaded to Rayyan for screening [13]. Study selection was performed independently by two reviewers in two sequential stages. Initially, titles and abstracts were screened for potential relevance. Subsequently, the full texts of potentially eligible studies were reviewed in detail according to the predefined inclusion and exclusion criteria. Any discrepancies were resolved through discussion and consensus, with arbitration by a third reviewer when required.

### Eligibility criteria

Eligible studies were RCTs, including both parallel-group and crossover designs, that enrolled adult patients with DM across a broad spectrum of CAD presentations, including stable CAD or chronic coronary syndrome (CCS), acute coronary syndrome (ACS), unstable angina (UA), non–ST-elevation myocardial infarction (NSTEMI), and ST-elevation myocardial infarction (STEMI). Most studies included patients with type 2 DM treated with oral hypoglycemic agents and/or insulin, although some defined DM based on prior diagnosis, investigator-reported history, or standard glucose-based criteria. Eligible trials compared oral P2Y12 inhibitors, including ticagrelor, prasugrel, and clopidogrel, either head-to-head or versus placebo, typically on a background of aspirin therapy ranging from 81 to 325 mg/day. The most used regimens were ticagrelor 180 mg loading dose followed by 90 mg twice daily, prasugrel 60 mg loading dose followed by 10 mg once daily, and clopidogrel 300–600 mg loading dose followed by 75 mg once daily. Studies were excluded if they were non-randomized, observational, reviews, editorials, case reports, animal studies, duplicate publications, or lacked extractable data for the diabetic CAD population.

### Outcomes

The primary efficacy outcome was major adverse cardiovascular events (MACEs), defined as a composite of cardiovascular (CV) death, myocardial infarction (MI), and stroke. Secondary efficacy outcomes included CV death, all-cause mortality, MI, and stroke. Safety outcomes included dyspnea and major bleeding events, including Bleeding Academic Research Consortium (BARC) type 3–5 major bleeding, Thrombolysis In Myocardial Infarction (TIMI) major bleeding, and Platelet Inhibition and Patient Outcomes (PLATO) major bleeding, according to trial-specific definitions. Additional pharmacodynamic outcomes included platelet reactivity and platelet aggregation inhibition.

### Data extraction

Data extraction was conducted independently by two reviewers using a standardized, predefined data extraction form. The extracted data included study characteristics, patient demographic characteristics, and reported outcomes. For quantitative synthesis, dichotomous outcomes were collected as the number of events and the total number of participants in each group, whereas continuous outcomes were extracted as means and standard deviations (SDs). When continuous data were reported as medians with interquartile ranges (IQRs) or in other summary formats, appropriate statistical methods were applied to estimate the mean and SD. Any discrepancies between the reviewers were resolved through discussion.

### Risk of bias assessment

The risk of bias of included RCTs was assessed independently by two reviewers using the Cochrane Risk of Bias 2 (RoB-2) tool [14], which evaluates bias across five domains: randomization process, deviations from intended interventions, missing outcome data, measurement of outcomes, and selection of reported results. Each study was classified as having low risk of bias, some concerns, or high risk of bias. Disagreements between reviewers were resolved by consensus.

### Statistical analysis and heterogeneity assessment

All statistical analyses were performed using R software (version 4.5.1; R Foundation for Statistical Computing, Vienna, Austria) according to the intention-to-treat principle, whereby all randomized patients were included in the analysis. An NMA was conducted using the netmeta package to synthesize direct and indirect evidence across ticagrelor, prasugrel, clopidogrel, and placebo within a unified analytical framework. Treatment effects were estimated using random-effects models, accounting for both within-study and between-study variability while preserving the correlation structure of multi-arm trials. A continuity correction of 0.5 was applied for studies reporting zero events in one treatment arm. Dichotomous outcomes were expressed as risk ratios (RRs) with 95% confidence intervals (CIs), whereas continuous outcomes were expressed as mean differences (MDs) with 95% CIs. In the overall NMA, placebo was used as the reference treatment when connected within the network. In subgroup analyses, clopidogrel was used as the reference treatment in the ACS network because placebo-controlled evidence was unavailable, whereas placebo remained the reference treatment in the stable CAD network. Global heterogeneity within the network was quantified using τ^2^ and I^2^ statistics, with I^2^ values of < 25%, 25% to 50%, and > 50% interpreted as low, moderate, and high heterogeneity, respectively. Inconsistency between direct and indirect evidence was assessed using the design-by-treatment interaction model. Local inconsistency was further examined using node-splitting or side-splitting methods when both direct and indirect evidence were available for the same comparison. Pairwise meta-analyses were also performed using the meta package. Random-effects models were applied throughout because of the anticipated clinical and methodological heterogeneity among the included trials. Statistical heterogeneity in pairwise analyses was assessed using Cochran’s Q test and quantified with the I^2^ statistic. A sensitivity analysis of clinical outcomes was performed by excluding pharmacodynamic-only studies because these trials were generally short-term, frequently used crossover designs, and assessed platelet reactivity rather than clinical outcomes. This analysis was performed to evaluate whether inclusion of pharmacodynamic-only studies influenced the direction, magnitude, or interpretation of the main clinical findings. Publication bias was evaluated for outcomes including at least 10 studies using funnel plot inspection and Egger’s regression test. Results were presented using forest plots, league tables, and network diagrams. Treatment hierarchy for efficacy and safety outcomes was assessed using P-scores, which range from 0 to 1. Higher P-score values indicate a greater probability that a treatment ranks favorably for a given efficacy or safety outcome, whereas lower values indicate a less favorable relative ranking. However, P-scores were interpreted as supportive ranking measures and not as independent evidence of treatment superiority. All tests were two-sided, and statistical significance was defined as a 95% CI that did not include the null value or a P value < 0.05.

## Results

### Literature search

**Figure 1:**
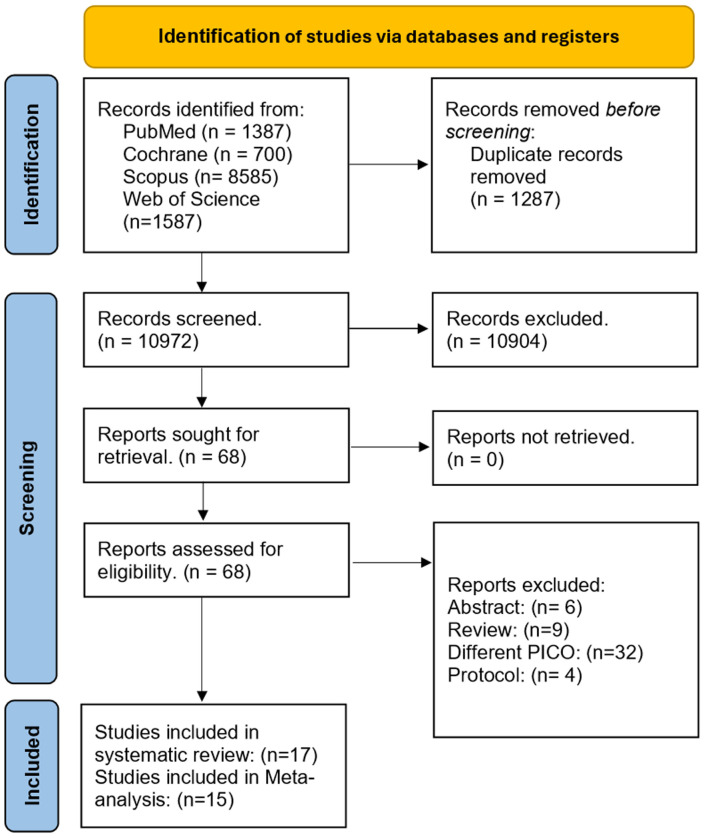
PRISMA flow diagram of study selection.

The literature search identified a total of 12,259 records from electronic databases. After removal of 1,287 duplicate records, 10,972 records remained for title and abstract screening. Of these, 10,904 records were excluded, leaving 68 full-text reports to be assessed in detail based on the predefined PICO criteria. Ultimately, 17 studies were included in the systematic review [2, 9, 15–29], of which 15 were included in the meta-analysis [2, 9, 15–27]. The study selection process is illustrated in the PRISMA flow diagram (Fig. 1).

### Characteristics of the included studies

Seventeen RCTs, comprising 40,919 participants, were included in the systematic review and meta-analysis. The included studies investigated various P2Y12 inhibitor–based therapeutic strategies in patients with diabetes and CAD, generally administered on a background of aspirin therapy (81–325 mg/day). Of the 17 studies, six compared ticagrelor versus clopidogrel, six compared ticagrelor versus prasugrel, three compared prasugrel versus clopidogrel, and two compared ticagrelor versus placebo. The weighted mean age of the study population was 64.8 years. Follow-up duration varied substantially across studies, ranging from short-term in-hospital or pharmacodynamic assessments to 39.9 months. After standardization of follow-up duration, the weighted mean follow-up was 29.9 months, and the weighted median follow-up was 33.0 months. Detailed summaries and baseline characteristics of the included studies are presented in Supplementary Material 1 (cr.elmerpub.com).

### Risk of bias assessment

Risk of bias was assessed for the included RCTs using the RoB-2 tool, which demonstrated that most studies were at low risk of bias, while three studies showed some concerns (Supplementary Material 1, cr.elmerpub.com).

### Primary effectiveness: MACEs

**Figure 2:**
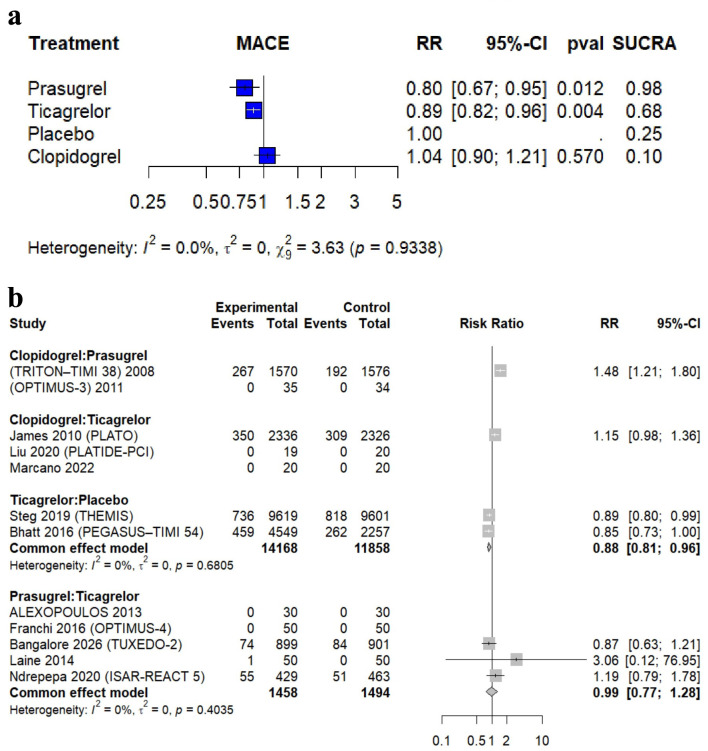
Forest plot of major adverse cardiovascular events (MACEs). (a) Network meta-analysis. (b) Pairwise meta-analysis.

In the NMA, 12 trials involving 36,934 patients reported 3,658 (9.9%) MACEs. Compared with placebo, both prasugrel and ticagrelor were associated with significant reductions in MACE, with pooled RR of 0.80 (95% CI, 0.67–0.95) and 0.89 (95% CI, 0.82–0.96), respectively. In contrast, clopidogrel was not associated with a significant reduction in MACE (RR, 1.04 (95% CI, 0.90–1.21)) (Fig. 2a). Direct pairwise comparison analyses showed that clopidogrel was associated with a significantly higher risk of MACE than prasugrel (RR, 1.48 (95% CI, 1.21–1.80)). Additionally, ticagrelor did not differ significantly from either clopidogrel or prasugrel; however, it was associated with a significant reduction in MACE compared with placebo (RR, 0.88 (95% CI, 0.81–0.96; Fig. 2b). In subgroup analyses according to clinical presentation, prasugrel significantly reduced MACE in patients with ACS compared with clopidogrel (RR, 0.78; 95% CI, 0.63–0.96; P = 0.018), while ticagrelor showed a non-significant trend toward lower MACE. In the stable CAD subgroup, ticagrelor significantly reduced MACE compared with placebo (RR, 0.89; 95% CI, 0.82–0.96; P = 0.004), whereas prasugrel showed a non-significant trend toward benefit. Clopidogrel also showed no significant difference compared with placebo. According to SUCRA rankings, prasugrel was the highest-ranked treatment for reducing MACE incidence (Supplementary Material 1, cr.elmerpub.com). We assessed publication bias using a funnel plot, which appeared largely symmetrical, suggesting no strong evidence of publication bias or small-study effects (Supplementary Material 1, cr.elmerpub.com).

### Secondary efficacy outcomes

#### CV mortality

**Figure 3:**
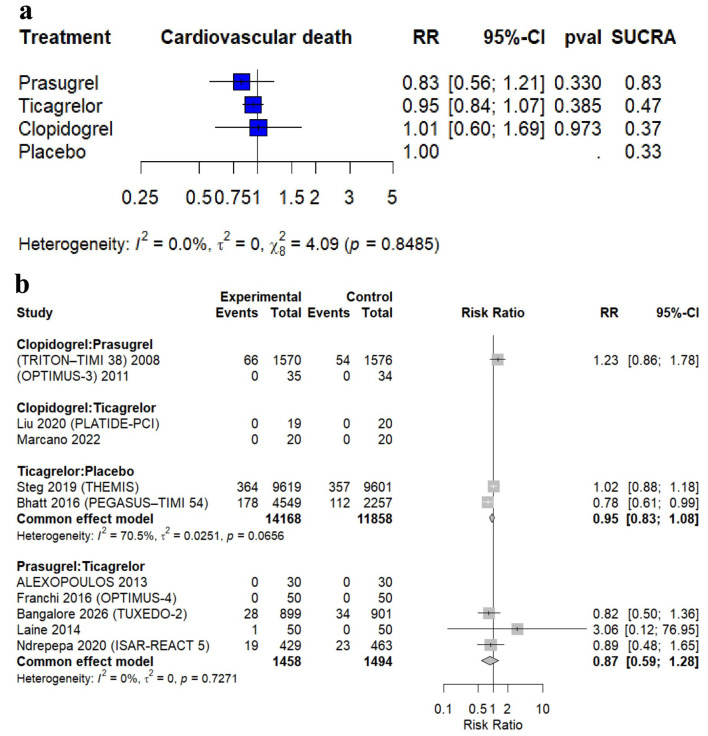
Forest plot of cardiovascular (CV) mortality. (a) Network meta-analysis. (b) Pairwise meta-analysis.

In the NMA, 11 trials involving 32,272 patients reported 1,236 CV mortality events (3.8%). Compared with placebo, the pooled analyses showed no significant reduction in CV mortality with any of the evaluated P2Y12 inhibitors. The pooled RR was 0.83 (95% CI, 0.56–1.21) for prasugrel, 0.95 (95% CI, 0.84–1.07) for ticagrelor, and 1.01 (95% CI, 0.60–1.69) for clopidogrel (Fig. 3a). Direct pairwise comparison analyses showed no significant differences in CV mortality among all treatment groups (Fig. 3b). By visual inspection, the funnel plot appears slightly asymmetrical, suggesting possible small-study effects or publication bias (Supplementary Material 1, cr.elmerpub.com).

#### All-cause mortality

**Figure 4:**
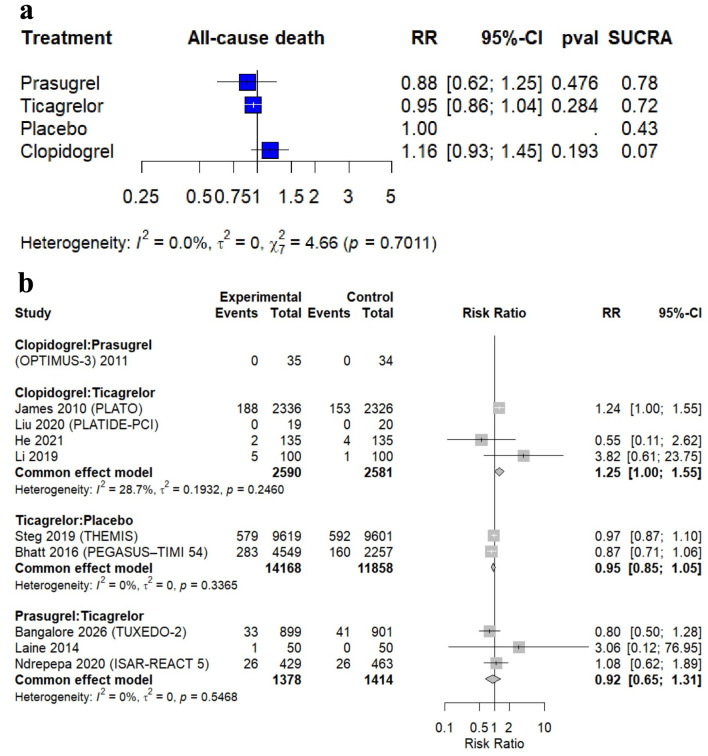
Forest plot of all-cause mortality. (a) Network meta-analysis. (b) Pairwise meta-analysis.

In the NMA, 10 trials involving 34,058 patients reported 2,094 all-cause mortality events (6.14%). Compared with placebo, the pooled analyses showed no statistically significant differences in all-cause mortality for any of the evaluated P2Y12 inhibitors. The pooled RR was 0.88 (95% CI, 0.62–1.25) for prasugrel, 0.95 (95% CI, 0.86–1.04) for ticagrelor, and 1.16 (95% CI, 0.93–1.45) for clopidogrel (Fig. 4a). Direct pairwise comparison analyses showed no significant differences in all-cause mortality among all treatment groups (Fig. 4b). By visual inspection, the funnel plot appears slightly asymmetrical, suggesting possible small-study effects or potential publication bias (Supplementary Material 1, cr.elmerpub.com).

#### Myocardial infarction

In the NMA, 14 trials involving 37,404 patients reported 1,880 MI events (5.02%). Compared with placebo, both prasugrel and ticagrelor were associated with significant reductions in MI, with pooled RRs of 0.69 (95% CI, 0.54–0.88) and 0.86 (95% CI, 0.76–0.97), respectively. In contrast, clopidogrel was not associated with a significant reduction in MI (RR, 1.02 (95% CI, 0.82–1.26)) (Supplementary Material 1, cr.elmerpub.com). Direct pairwise comparison analyses showed that clopidogrel was associated with a significantly higher risk of MI than prasugrel (RR, 1.70 (95% CI, 1.35–2.15)). Furthermore, no significant differences were observed between ticagrelor and either clopidogrel or prasugrel. However, ticagrelor was associated with a significantly lower risk of MI compared with placebo (RR, 0.85 (95% CI, 0.75–0.97)) (Supplementary Material 1, cr.elmerpub.com). By visual inspection, the funnel plot appears largely symmetrical around the pooled effect, suggesting no strong evidence of publication bias (Supplementary Material 1, cr.elmerpub.com).

#### Stroke

In the NMA, 10 trials including 29,357 patients reported 565 stroke events (1.92%). Compared with placebo, ticagrelor significantly reduced the risk of stroke (RR, 0.79 (95% CI, 0.67–0.93)), whereas prasugrel and clopidogrel showed no statistically significant effect on stroke risk (Supplementary Material 1, cr.elmerpub.com). Direct pairwise comparison analyses showed that ticagrelor was associated with a statistically significant reduction in stroke compared with placebo (RR, 0.78 (95% CI, 0.66–0.93)), whereas all other treatment comparisons showed no statistically significant differences (Supplementary Material 1, cr.elmerpub.com). The funnel plot appears slightly asymmetrical, suggesting potential small-study effects or publication bias (Supplementary Material 1, cr.elmerpub.com).

### Secondary safety outcomes

#### BARC 3–5 major bleeding

**Figure 5:**
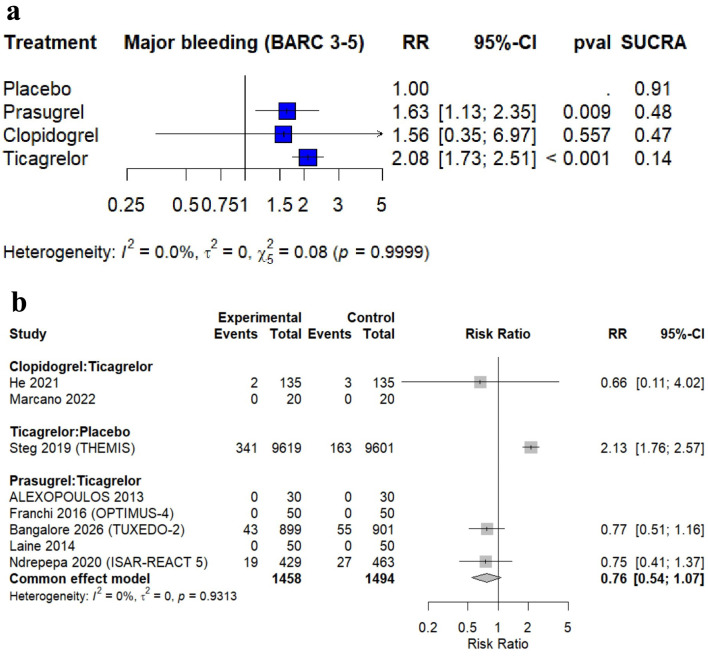
Forest plot of Bleeding Academic Research Consortium grade 3–5 major bleeding (BARC 3–5). (a) Network meta-analysis. (b) Pairwise meta-analysis.

In the NMA, eight trials including 22,482 patients reported 653 (2.9%) BARC 3–5 major bleeding events. Compared with placebo, both ticagrelor and prasugrel were associated with a statistically significant increase in the risk of major bleeding, with pooled RRs of 2.08 (95% CI, 1.73–2.51) and 1.63 (95% CI, 1.13–2.35), respectively, whereas clopidogrel showed no statistically significant difference versus placebo (Fig. 5a). Direct pairwise comparison analyses showed that ticagrelor was associated with a statistically significant increase in major bleeding compared with placebo (RR, 2.13 (95% CI, 1.76–2.57)), whereas all other treatment comparisons showed no statistically significant differences (Fig. 5b).

#### TIMI major bleeding

In the NMA, seven trials including 34,043 patients reported 909 (2.67%) TIMI major bleeding events. Compared with placebo, ticagrelor, prasugrel, and clopidogrel were all associated with a statistically significant increase in the risk of TIMI major bleeding, with pooled RRs of 2.15 (95% CI, 1.75–2.66), 2.29 (95% CI, 1.38–3.81), and 2.42 (95% CI, 1.82–3.21), respectively (Supplementary Material 1, cr.elmerpub.com). Direct pairwise comparison analyses showed that ticagrelor was associated with a statistically significant increase in TIMI major bleeding compared with placebo (RR, 2.21 (95% CI, 1.79–2.73)), whereas all other treatment comparisons showed no statistically significant differences (Supplementary Material 1, cr.elmerpub.com).

#### PLATO major bleeding

In the NMA, four trials including 23,961 patients reported 1,047 (4.3) PLATO major bleeding events. Compared with placebo, both ticagrelor and clopidogrel were associated with a statistically significant increase in the risk of PLATO major bleeding, with pooled RRs of 2.13 (95% CI, 1.75–2.59) and 2.25 (95% CI, 1.76–2.88), respectively (Supplementary Material 1, cr.elmerpub.com). Direct pairwise comparison analyses showed that ticagrelor was associated with a statistically significant increase in PLATO major bleeding compared with placebo (RR, 2.17 (95% CI, 1.78–2.65)), whereas the other treatment comparisons showed no statistically significant differences (Supplementary Material 1, cr.elmerpub.com).

#### Dyspnea

In the NMA, six trials involving 19,659 patients reported 2,781 (14.1%) dyspnea events. Compared with placebo, ticagrelor was associated with a statistically significant increase in the risk of dyspnea (RR, 2.92 (95% CI, 2.69–3.17)), whereas prasugrel and clopidogrel showed no statistically significant differences (Supplementary Material 1, cr.elmerpub.com). Direct pairwise comparison analyses showed that ticagrelor was associated with a statistically significant increase in dyspnea compared with placebo, clopidogrel, and prasugrel (Supplementary Material 1, cr.elmerpub.com).

### Pharmacodynamic outcomes

#### Platelet reactivity (PRU – VerifyNow)

In comparison with clopidogrel, both ticagrelor and prasugrel were associated with statistically significant reductions in PRU, with MDs of −116.25 (95% CI, −158.59 to −73.91) and −82.89 (95% CI, −142.51 to −23.27), respectively. In comparison with ticagrelor, prasugrel showed no statistically significant difference, whereas clopidogrel was associated with significantly higher PRU (MD, 116.25 (95% CI, 73.91–158.59)). In comparison with prasugrel, ticagrelor showed no statistically significant difference, whereas clopidogrel was associated with significantly higher PRU (MD, 82.89 (95% CI, 23.27–142.51)) (Supplementary Material 1, cr.elmerpub.com). Direct pairwise analyses showed that ticagrelor was associated with significantly lower PRU than clopidogrel (MD, −117.95 (95% CI, −179.62 to −56.29)) and prasugrel (MD, −33.59 (95% CI, −51.76 to −15.43)) (Supplementary Material 1, cr.elmerpub.com).

#### Percent platelet inhibition (%)

In comparison with clopidogrel, both ticagrelor and prasugrel were associated with statistically significant increases in percent platelet inhibition, with MDs of 54.85 (95% CI, 29.28–80.43) and 51.34 (95% CI, 20.47–82.20), respectively. In comparison with ticagrelor, prasugrel showed no statistically significant difference, whereas clopidogrel was associated with significantly lower platelet inhibition (MD, −54.85 (95% CI, −80.43 to −29.28)). In comparison with prasugrel, ticagrelor showed no statistically significant difference, whereas clopidogrel was associated with significantly lower platelet inhibition (MD, −51.34 (95% CI, −82.20 to −20.47)) (Supplementary Material 1, cr.elmerpub.com). Direct pairwise analyses showed that ticagrelor was associated with significantly higher platelet inhibition than clopidogrel (MD, 49.41 (95% CI, 21.53–77.29)) and prasugrel (MD, 14.00 (95% CI, 3.78–24.22)), whereas prasugrel was also associated with significantly higher platelet inhibition than clopidogrel (MD, 61.60 (95% CI, 53.15–70.05)) (Supplementary Material 1, cr.elmerpub.com).

#### Sensitivity analysis

We conducted a sensitivity analysis excluding pharmacodynamic-only studies. The results were broadly consistent with the primary analysis. Prasugrel and ticagrelor remained associated with lower risks of MACE and MI compared with placebo, and ticagrelor remained associated with a lower risk of stroke. No significant differences were observed for cardiovascular or all-cause mortality. For safety outcomes, ticagrelor and prasugrel continued to show increased bleeding risks, and ticagrelor remained associated with a higher risk of dyspnea. Overall, excluding pharmacodynamic-only studies did not materially change the direction, magnitude, or interpretation of the main findings (Supplementary Material 1, cr.elmerpub.com).

#### Ranking of treatment strategies

Based on P-score ranking, prasugrel was the highest-ranked treatment for reducing MACE (0.969), MI (0.993), CV mortality (0.825), and all-cause mortality (0.782), whereas ticagrelor was the highest-ranked treatment for stroke prevention (0.736) (Supplementary Material 1, cr.elmerpub.com). For safety outcomes related to bleeding, placebo was consistently ranked as the safest strategy, with the highest P-scores for BARC 3–5 major bleeding (0.906), TIMI major bleeding (1.000), and PLATO major bleeding (1.000). Among the active comparators, clopidogrel ranked slightly more favorably than prasugrel for BARC 3–5 bleeding (0.483 vs. 0.473), while ticagrelor ranked more favorably for TIMI major bleeding (0.494) and PLATO major bleeding (0.384) (Supplementary Material 1, cr.elmerpub.com). Regarding dyspnea, prasugrel had the most favorable ranking (0.719), whereas ticagrelor had the lowest P-score (0.013) (Supplementary Material 1, cr.elmerpub.com).

#### Network consistency

The node-splitting method did not detect significant disagreement between direct and indirect evidence (Supplementary Material 1, cr.elmerpub.com). The Egger regression test did not demonstrate evidence of significant publication bias across all evaluated outcomes (all P > 0.05) (Supplementary Material 1, cr.elmerpub.com).

## Discussion

This systematic review and NMA of 17 RCTs encompassing 40,919 participants with CAD and DM provides a comprehensive hierarchical assessment of P2Y12 inhibitors. Our analysis suggests that prasugrel and ticagrelor were associated with more favorable ischemic outcomes, particularly MACE and MI, compared with clopidogrel and placebo. In contrast, more potent P2Y12 inhibition was associated with a higher bleeding risk. Prasugrel showed the most favorable profile for MACE and MI based on the main effect estimates and treatment ranking analysis; however, these rankings should be interpreted as supportive rather than definitive evidence of treatment superiority. Although ticagrelor showed a favorable effect on stroke, no significant reductions in CV mortality or all-cause mortality were observed across treatment strategies.

The exploratory subgroup analysis by clinical presentation provided additional clinical context. Among patients with ACS, prasugrel significantly reduced MACE compared with clopidogrel, whereas ticagrelor showed a non-significant trend toward benefit. In the stable CAD subgroup, ticagrelor significantly reduced MACE compared with placebo, while prasugrel and clopidogrel did not show statistically significant differences. According to SUCRA ranking, prasugrel appeared to be the most favorable treatment for reducing MACE. The sensitivity analysis excluding pharmacodynamic-only studies yielded results broadly consistent with the primary analysis, supporting the robustness of the main findings and indicating that the conclusions were not primarily driven by short-term pharmacodynamic trials. Our study focuses on patients with CAD and DM, as they may have enhanced platelet reactivity with increased P2Y12 receptor expression. Recent research studies have shown that individuals with type 2 diabetes exhibit fourfold higher P2Y12 receptor expression than healthy subjects [30, 31]. This enhancement may be due to multiple mechanisms, including increased platelet turnover leading to more immature circulating platelets, increased glycoprotein IIb/IIIa receptor density, and dysregulation of platelet receptor density and signaling ([32]. These mechanisms may explain the limited effectiveness of standard antiplatelet therapies, such as clopidogrel. In addition, previous studies showed that 40% lower exposure to clopidogrel’s active metabolite in patients with DM is the predominant mechanism of impaired response [30, 32], and this can result from decreased gastrointestinal absorption, increased prodrug hydrolysis to inactive metabolites by carboxylesterase 1, reduced hepatic cytochrome P450 activity, and increased active metabolite hydrolysis [31, 33]. Prasugrel is a third-generation thienopyridine that undergoes more efficient and consistent metabolic activation, resulting in more potent and predictable platelet inhibition. Ticagrelor is a reversible direct-acting P2Y12 inhibitor that bypasses metabolic activation altogether and achieves rapid and sustained platelet inhibition [34]. These mechanistic advantages are reflected in significantly lower platelet reactivity units and higher percentage platelet inhibition observed with prasugrel and ticagrelor compared with clopidogrel. Although P2Y12 inhibitors are associated with a reduction in MACE and MI rates, they showed an insignificant reduction in mortality rates, and the cause of that may be due to the lower absolute number of events for mortality, which limits statistical power to detect differences; also, risks between non-cardiovascular mortality and bleeding-related complications may attenuate net survival benefits.

Our findings showed that bleeding risk (BARC 3–5 and TIMI major bleeding) was higher in prasugrel and ticagrelor compared with placebo. However, these findings were consistent with pharmacologic potency and have been well documented in landmark trials. In TRITON-TIMI 38, prasugrel demonstrated a 32% increase in major bleeding compared to clopidogrel (2.4% vs. 1.8%; P = 0.03), with a particularly concerning increase in fatal bleeding (0.4% vs. 0.1%; P = 0.002). In PLATO, ticagrelor showed similar overall major bleeding rates to clopidogrel (11.6% vs. 11.2%, P = 0.43) but significantly increased major bleeding (4.5% vs. 3.8%, P = 0.02) and non-procedure-related major bleeding (3.1% vs. 2.3%, P = 0.05). The rapid onset and more potent platelet inhibition achieved by ticagrelor and prasugrel are speculated to be responsible for these higher bleeding rates [35, 36]. An NMA of 12 RCTs showed that both prasugrel and ticagrelor significantly increase major bleeding compared to clopidogrel, with no significant difference between the two potent agents [37]. Our findings were consistent with the recently published TUXEDO-2 trial, which specifically evaluated ticagrelor versus prasugrel in 1,850 patients with diabetes and multivessel coronary disease undergoing percutaneous coronary intervention. Ticagrelor failed to meet noninferiority criteria for the primary composite endpoint of death, MI, stroke, or major bleeding (11.8% vs. 9.4%; P = 0.10) [20]. Notably, prasugrel demonstrated particular advantages in two high-risk subgroups: patients with diabetes duration < 5 years and those classified as high bleeding risk. This finding contrasts with the ISAR-REACT 5 diabetes subgroup analysis, where ticagrelor and prasugrel showed comparable efficacy in diabetic patients. However, ISAR-REACT 5’s diabetes cohort represented only 22% of participants, whereas TUXEDO-2 enrolled exclusively diabetic patients [20, 38].

We can summarize the strengths of this study as follows. To our knowledge, this is the first NMA comparing the efficacy and safety of P2Y12 inhibitors in patients with CAD and DM. We include only RCTs with a large, aggregated sample size exceeding 30,000 patients. We conducted both direct and indirect evidence to enhance the precision and reliability of effect estimates. Additionally, the inclusion of pharmacodynamic outcomes provides mechanistic insights that support clinical findings. The absence of significant publication bias across most outcomes further strengthens the validity of the results.

### Limitations

Several limitations should be acknowledged. First, the included trials differed in important clinical and methodological aspects, including clinical presentation, acute versus stable CAD, treatment duration, and definitions of DM. Second, outcome definitions were not fully consistent across trials, particularly for bleeding endpoints, which may limit direct comparability across studies. Third, as with all NMAs, the validity of the findings depends on the transitivity assumption, which may have been affected by unmeasured differences in trial populations, study designs, treatment protocols, and follow-up durations. Fourth, the choice of reference treatment differed according to the available evidence within each network. Placebo was used only when a placebo arm was available and connected to the network; otherwise, clopidogrel was used as the standard active comparator. Fifth, the lack of platelet function studies represents a major limitation of this analysis, as most included trials did not systematically assess platelet function, report platelet reactivity measurements, or provide genetic testing related to clopidogrel metabolism. As a result, we could not determine whether platelet function was adequately inhibited by clopidogrel in individual patients. This is an important methodological limitation, particularly because patients with DM often have increased platelet reactivity and impaired responsiveness to clopidogrel. Therefore, the clopidogrel arms may have included a mixture of responders and non-responders, potentially influencing the comparative efficacy estimates and partly accounting for the less favorable outcomes observed with clopidogrel. In addition, the absence of patient-level data limited our ability to perform detailed subgroup analyses according to glycemic control, duration of diabetes, insulin use, renal function, genetic profile, baseline platelet reactivity, or individualized ischemic and bleeding risk. Finally, the relatively short follow-up duration in some trials may limit conclusions regarding long-term efficacy, safety, and net clinical benefit.

Future research should focus on addressing these gaps. Large, adequately powered randomized trials specifically targeting diabetic populations are needed to confirm the optimal choice of P2Y12 inhibitor. Studies evaluating long-term outcomes, including mortality and net clinical benefit, are particularly important. Additionally, investigations into personalized antiplatelet strategies, incorporating platelet function testing or genetic profiling, may help optimize therapy. The role of de-escalation strategies and combination therapies in balancing ischemic and bleeding risks also warrants further exploration.

### Conclusion

This NMA indicates that prasugrel and ticagrelor offer enhanced protection against ischemic events relative to clopidogrel in individuals with CAD and DM. Prasugrel exhibited the most significant reduction in MACE and MI, whereas ticagrelor demonstrated efficacy in stroke prevention. These advantages, however, are tempered by an elevated bleeding risk, thereby underscoring the necessity of personalized treatment decisions. Although no substantial mortality benefit was identified. These findings highlight the need for meticulous risk stratification and additional research to optimize antiplatelet strategies within this intricate patient population.

## Supplementary Material

Suppl 1 Supplementary files to this study.

## Data Availability

All data analyzed during this study are included in the published articles and supplementary materials of the included studies. Additional extracted data are available from the corresponding author upon reasonable request.
