# Supplementary material for: Comparative Efficacy and Safety of P2Y12 Inhibitors in Patients With Diabetes Mellitus and Coronary Artery Disease: A Systematic Review and Network Meta-Analysis of Randomized Controlled Trials
Source: Cardiol Res. 2026 Jul 15;17(4):347–59. doi: 10.14740/cr2254 (PMC13411678; doi:10.14740/cr2254)
Supplement: Suppl 1 — Supplementary files to this study. [file cr-17-04-347-s001.docx]

**Supplementary Materials**

|  | **Page** |
| --- | --- |
| **Supplementary Table 1:** Detailed search strategy for each database | **3** |
| **Supplementary Table 2:** Net league table of Efficacy outcomes | **4** |
| **Supplementary Table 3:** Net league of safety outcomes | **5** |
| **Supplementary Table 4**. Net league table of pharmacodynamic outcomes | **6** |
| **Figure S1.** Risk of bias assessment of included randomized controlled trials using the Risk of Bias 2 (RoB-2) tool. | **7** |
| **Figure S2.** Subgroup analysis of major adverse cardiovascular events (MACE) stratified by coronary artery disease presentation. (A) Acute coronary syndrome. (B) Stable coronary artery disease | **8** |
| **Figure S3.** Funnel plot of major adverse cardiovascular events (MACE). | **9** |
| **Figure S4.** Network diagram of major adverse cardiovascular events (MACE). | **10** |
| **Figure S5.** Funnel plot of cardiovascular mortality (CV mortality). | **11** |
| **Figure S6.** Network diagram of cardiovascular mortality. | **12** |
| **Figure S7.** Funnel plot of all-cause mortality. | **13** |
| **Figure S8.** Network diagram of all-cause mortality. | **14** |
| **Figure S9.** Network meta-analysis forest plot of myocardial infarction (MI). | **15** |
| **Figure S10.** Pairwise meta-analysis forest plot of myocardial infarction (MI). | **16** |
| **Figure S11.** Funnel plot of myocardial infarction (MI). | **17** |
| **Figure S12.** Network diagram of myocardial infarction (MI). | **18** |
| **Figure S13.** Network meta-analysis forest plot of stroke. | **19** |
| **Figure S14.** Pairwise meta-analysis forest plot of stroke. | **20** |
| **Figure S15.** Funnel plot of stroke. | **21** |
| **Figure S16.** Network diagram of stroke. | **22** |
| **Figure S17.** Network diagram of major bleeding [BARC 3–5]. | **23** |
| **Figure S18.** Network meta-analysis forest plot of TIMI major bleeding. | **24** |
| **Figure S19.** Pairwise meta-analysis forest plot of TIMI major bleeding. | **25** |
| **Figure S20.** Network diagram of TIMI major bleeding. | **26** |
| **Figure S21.** Network meta-analysis forest plot of PLATO major bleeding. | **27** |
| **Figure S22.** Pairwise meta-analysis forest plot of PLATO major bleeding. | **28** |
| **Figure S23.** Network diagram of PLATO major bleeding. | **29** |
| **Figure S24.** Network meta-analysis forest plot of dyspnea. | **30** |
| **Figure S25.** Pairwise meta-analysis forest plot of dyspnea. | **31** |
| **Figure S26.** Network diagram of dyspnea. | **32** |
| **Figure S27.** Network meta-analysis forest plot of platelet reactivity | **33** |
| **Figure S28.** Pairwise meta-analysis forest plot of platelet reactivity | **34** |
| **Figure S29.** Network diagram of platelet reactivity | **35** |
| **Figure S30.** Network meta-analysis forest plot of percent platelet inhibition. | **36** |
| **Figure S31.** Pairwise meta-analysis forest plot of percent platelet inhibition. | **37** |
| **Figure S32.** Network diagram of percent platelet inhibition. | **38** |
| **Figure S33.** Sensitivity network meta-analysis for MACE after exclusion of pharmacodynamic-only studies. | **39** |
| **Figure S34.** Sensitivity network meta-analysis for myocardial infarction after exclusion of pharmacodynamic-only studies. | **40** |
| **Figure S35.** Sensitivity network meta-analysis for stroke after exclusion of pharmacodynamic-only studies. | **41** |
| **Figure S36.** Sensitivity network meta-analysis for cardiovascular mortality after exclusion of pharmacodynamic-only studies. | **42** |
| **Figure S37.** Sensitivity network meta-analysis for all-cause mortality after exclusion of pharmacodynamic-only studies. | **43** |
| **Figure S38.** Sensitivity network meta-analysis for BARC 3–5 major bleeding after exclusion of pharmacodynamic-only studies. | **44** |
| **Figure S39.** Sensitivity network meta-analysis for TIMI major bleeding after exclusion of pharmacodynamic-only studies. | **45** |
| **Figure S40.** Sensitivity network meta-analysis for PLATO major bleeding after exclusion of pharmacodynamic-only studies. | **46** |
| **Figure S41.** Sensitivity network meta-analysis for dyspnea after exclusion of pharmacodynamic-only studies. | **47** |
| **Figure S42.** Ranking of treatment strategies based on P-scores across efficacy outcomes. | **48** |
| **Figure S43.** Ranking of treatment strategies based on P-scores across safety outcomes. | **49** |
| **Supplementary Table 5.** Assessment of Consistency of the Network Meta-Analysis Model | **50** |
| **Supplementary Table 6.** Egger’s regression test | **51** |
| **Supplementary Table 7.** Summary of the Studies Included in This Review | **52** |
| **Supplementary Table 8.** Baseline Demographic Characteristics of the Study Population | **65** |

**Supplementary Table 1:** Detailed search strategy for each database

| Database | Search term | Filter | Results |
| --- | --- | --- | --- |
| PubMed | ("coronary artery disease" OR "CAD" OR "acute coronary syndrome" OR "ACS" OR "myocardial infarction" OR "MI" OR "unstable angina" OR "UA") AND ("diabetes mellitus" OR "DM" OR "diabetes" OR "diabetic") AND ("clopidogrel" OR "prasugrel" OR "ticagrelor" OR "P2Y12" OR "P2Y12 inhibitor") | All Filed | 1387 |
| Scopus | ("coronary artery disease" OR "CAD" OR "acute coronary syndrome" OR "ACS" OR "myocardial infarction" OR "MI" OR "unstable angina" OR "UA") AND ("diabetes mellitus" OR "DM" OR "diabetes" OR "diabetic") AND ("clopidogrel" OR "prasugrel" OR "ticagrelor" OR "P2Y12" OR "P2Y12 inhibitor") | Article title, abstract, keywords with article limited | 8,585 |
| Web of Science | ("coronary artery disease" OR "CAD" OR "acute coronary syndrome" OR "ACS" OR "myocardial infarction" OR "MI" OR "unstable angina" OR "UA") AND ("diabetes mellitus" OR "DM" OR "diabetes" OR "diabetic") AND ("clopidogrel" OR "prasugrel" OR "ticagrelor" OR "P2Y12" OR "P2Y12 inhibitor") | All Filed | 1,587 |
| Cochrane library | ("coronary artery disease" OR "CAD" OR "acute coronary syndrome" OR "ACS" OR "myocardial infarction" OR "MI" OR "unstable angina" OR "UA") AND ("diabetes mellitus" OR "DM" OR "diabetes" OR "diabetic") AND ("clopidogrel" OR "prasugrel" OR "ticagrelor" OR "P2Y12" OR "P2Y12 inhibitor") | All Filed | 700 |

| **Supplementary Table 2:** Net league table of Efficacy outcomes | | | |
| --- | --- | --- | --- |
| MACE | | | |
| Clopidogrel | . | 1.39 (1.17;1.65) | 1.13 (0.98;1.30) |
| 1.04 (0.90;1.21) | Placebo | . | 1.12 (1.04;1.22) |
| 1.31 (1.14;1.52) | 1.26 (1.05;1.50) | Prasugrel | 1.00 (0.79;1.25) |
| 1.17 (1.03;1.33) | 1.12 (1.04;1.22) | 0.90 (0.76;1.05) | Ticagrelor |
| CV mortality | | | |
| Clopidogrel | . | 1.22 (0.86; 1.73) | 1.03 (0.07;15.84) |
| 1.01 (0.60;1.69) | Placebo | . | 1.06 (0.93; 1.19) |
| 1.22 (0.86;1.73) | 1.21 (0.82;1.78) | Prasugrel | 0.87 (0.60; 1.26) |
| 1.06 (0.65;1.75) | 1.06 (0.93;1.19) | 0.87 (0.60;1.26) | Ticagrelor |
| All-cause death | | | |
| Clopidogrel | . | 0.97 (0.02;47.62) | 1.22 (1.00; 1.50) |
| 1.16 (0.93;1.45) | Placebo | . | 1.05 (0.96; 1.16) |
| 1.32 (0.89;1.95) | 1.14 (0.80;1.61) | Prasugrel | 0.93 (0.66; 1.30) |
| 1.22 (1.00;1.49) | 1.05 (0.96;1.16) | 0.93 (0.66;1.30) | Ticagrelor |
| MI | | | |
| Clopidogrel | . | 1.61 (1.30;1.98) | 1.09 (0.90;1.33) |
| 1.02 (0.82;1.26) | Placebo | . | 1.16 (1.03;1.32) |
| 1.47 (1.23;1.76) | 1.44 (1.13;1.84) | Prasugrel | 0.98 (0.72;1.33) |
| 1.18 (1.00;1.40) | 1.16 (1.03;1.32) | 0.81 (0.65;0.99) | Ticagrelor |
| Stroke | | | |
| Clopidogrel | . | 0.97 (0.02;47.62) | 1.50 (0.25; 8.90) |
| 1.10 (0.21;5.66) | Placebo | . | 1.27 (1.07; 1.50) |
| 1.33 (0.24;7.50) | 1.21 (0.58;2.54) | Prasugrel | 1.04 (0.50; 2.16) |
| 1.40 (0.28;7.12) | 1.27 (1.07;1.50) | 1.05 (0.51;2.16) | Ticagrelor |

| **Supplementary Table 3:** Net league of safety outcomes | | | |
| --- | --- | --- | --- |
| Major Bleeding BRAC 3-5 | | | |
| Clopidogrel | . | . | 0.75 (0.17;3.31) |
| 1.56 (0.35;6.97) | Placebo | . | 0.48 (0.40;0.58) |
| 0.96 (0.21;4.37) | 0.61 (0.43;0.88) | Prasugrel | 0.78 (0.57;1.07) |
| 0.75 (0.17;3.31) | 0.48 (0.40;0.58) | 0.78 (0.57;1.07) | Ticagrelor |
| TIMI Major Bleeding | | | |
| Clopidogrel | . | 1.05 (0.69; 1.62) | 1.12 (0.93; 1.36) |
| 2.42 (1.82;3.21) | Placebo | . | 0.46 (0.38; 0.57) |
| 1.05 (0.69;1.61) | 0.44 (0.26;0.73) | Prasugrel | 1.00 (0.02;49.43) |
| 1.12 (0.93;1.36) | 0.46 (0.38;0.57) | 1.06 (0.67;1.69) | Ticagrelor |
| PLATO major bleeding | | |  |
| Clopidogrel | . | 1.06 (0.91;1.23) |  |
| 2.25 (1.76;2.88) | Placebo | 0.47 (0.39;0.57) |  |
| 1.06 (0.91;1.23) | 0.47 (0.39;0.57) | Ticagrelor |  |
| Dyspnea | | | |
| Clopidogrel | . | . | 0.31 (0.10; 1.02) |
| 0.92 (0.28; 2.97) | Placebo | . | 0.34 (0.32; 0.37) |
| 1.09 (0.22; 5.36) | 1.19 (0.41; 3.50) | Prasugrel | 0.29 (0.10; 0.84) |
| 0.31 (0.10; 1.02) | 0.34 (0.32; 0.37) | 0.29 (0.10; 0.84) | Ticagrelor |

| **Supplementary Table 4**. Net league table of pharmacodynamic outcomes | | |
| --- | --- | --- |
| Platelet reactivity (PRU – VerifyNow) | | |
| Clopidogrel | . | 116.25 (73.91;158.59) |
| 82.89 (23.27;142.51) | Prasugrel | 33.36 (-8.61; 75.33) |
| 116.25 (73.91;158.59) | 33.36 (-8.61; 75.33) | Ticagrelor |
| Percent platelet inhibition (%) | | |
| Clopidogrel | -61.60 (-101.20; -22.00) | -49.46 (-78.17; -20.75) |
| -51.34 (-82.20; -20.47) | Prasugrel | -14.00 ( -54.02; 26.02) |
| -54.85 (-80.43; -29.28) | -3.52 (-34.49; 27.45) | Ticagrelor |

**Figure S1.** Risk of bias assessment of included randomized controlled trials using the Risk of Bias 2 (RoB-2) tool.


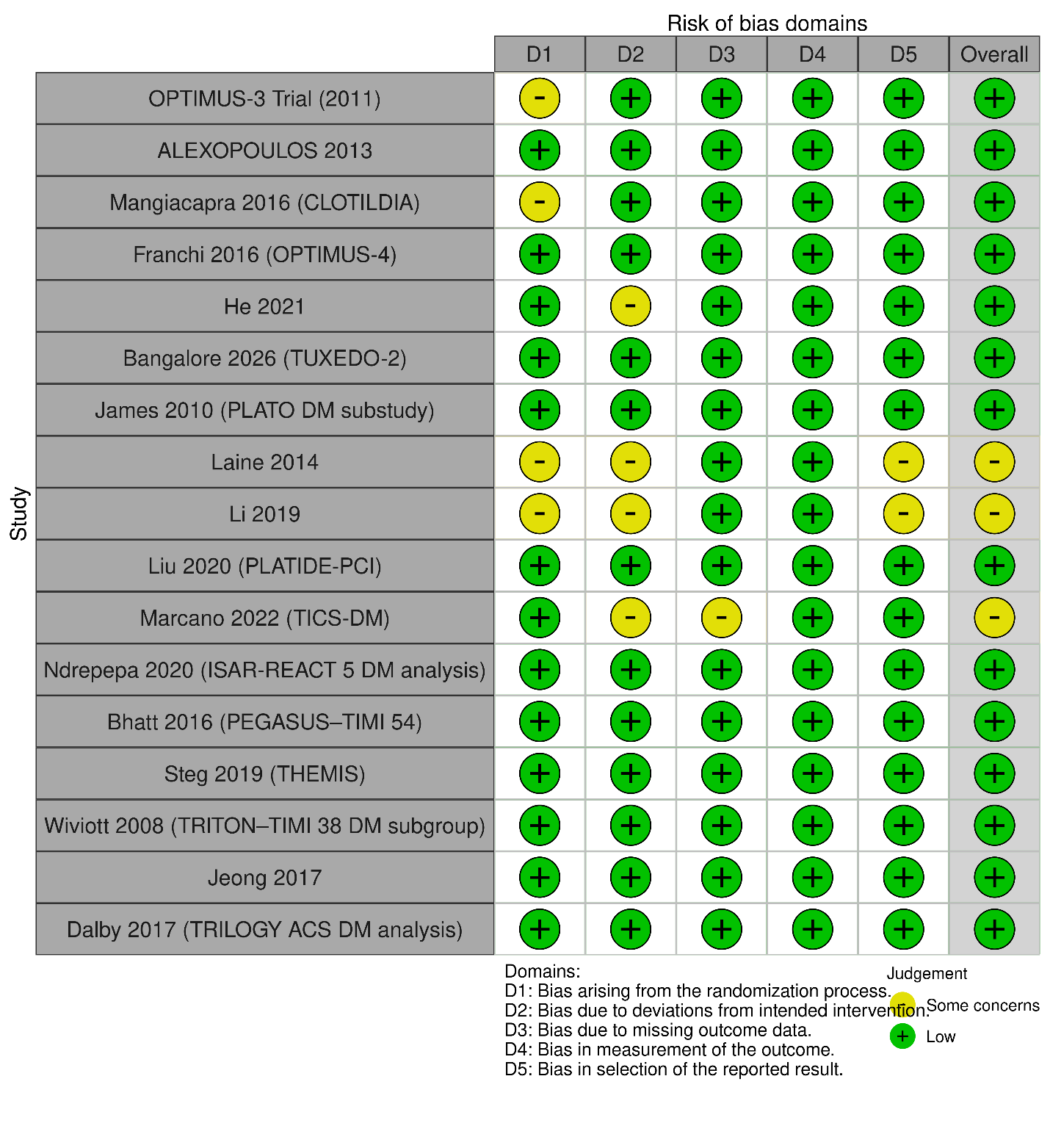


**Figure S2.** Subgroup analysis of major adverse cardiovascular events (MACE) stratified by coronary artery disease presentation. (A) Acute coronary syndrome. (B) Stable coronary artery disease.
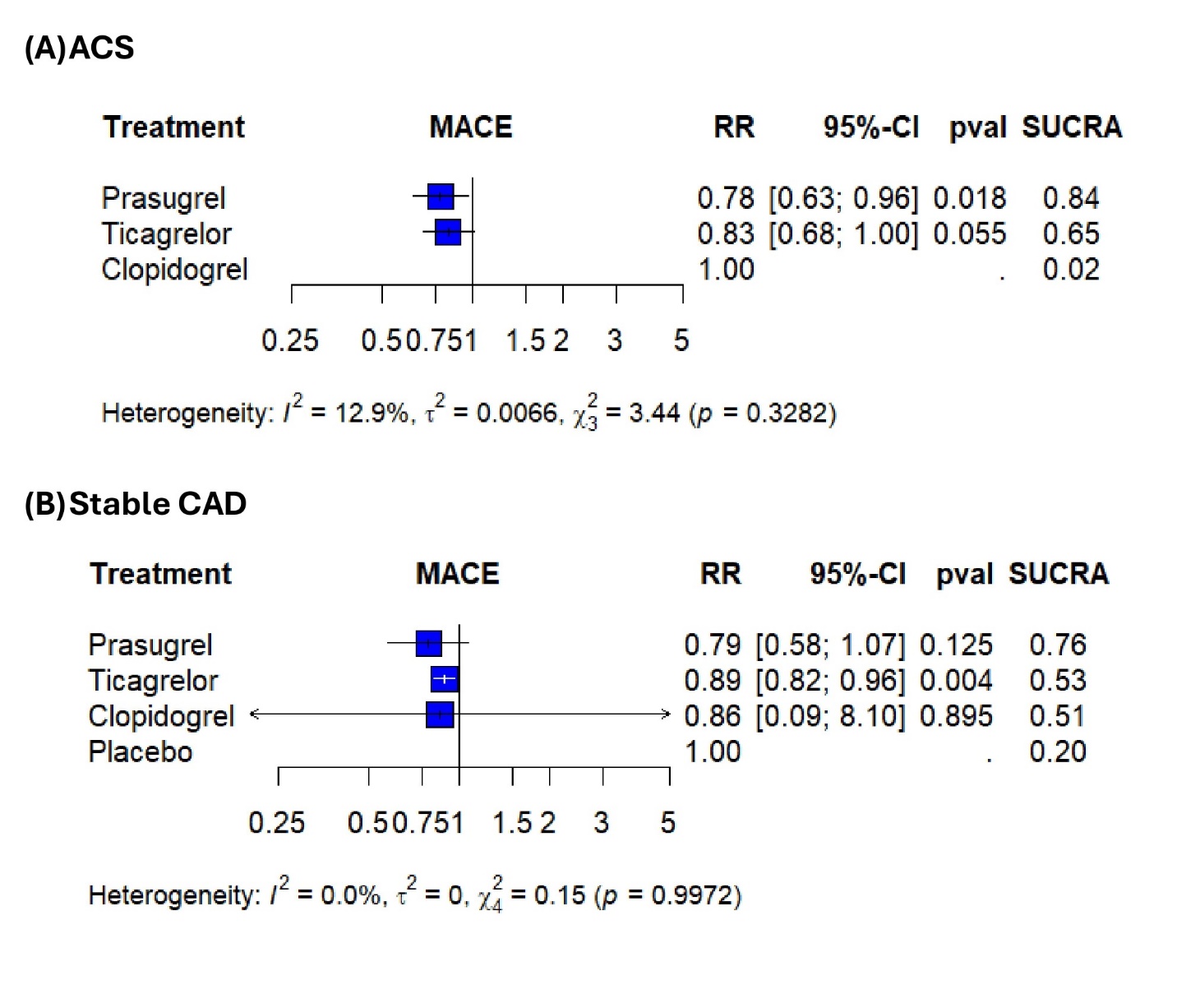


**Figure S3.** Funnel plot of major adverse cardiovascular events (MACE).


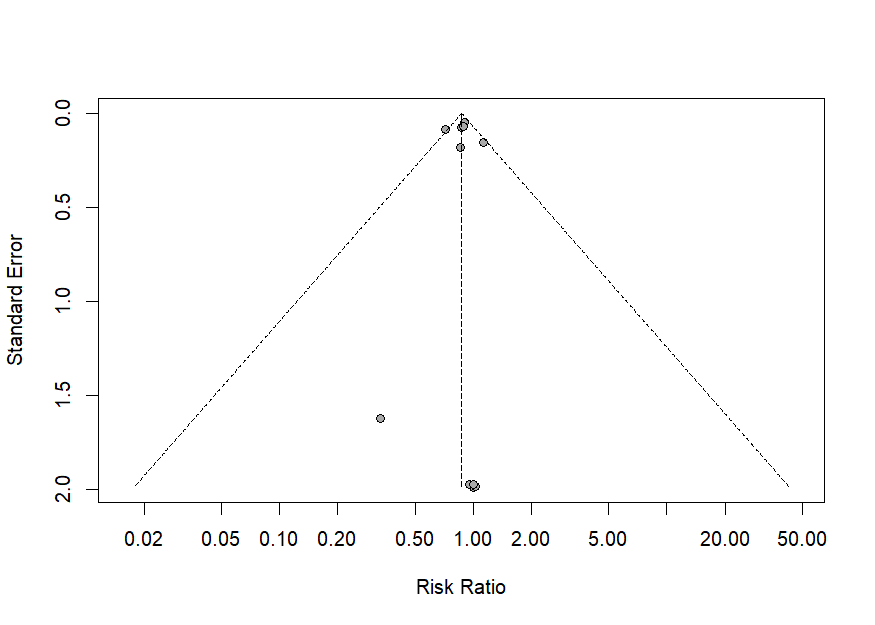


**Figure S4.** Network diagram of major adverse cardiovascular events (MACE).


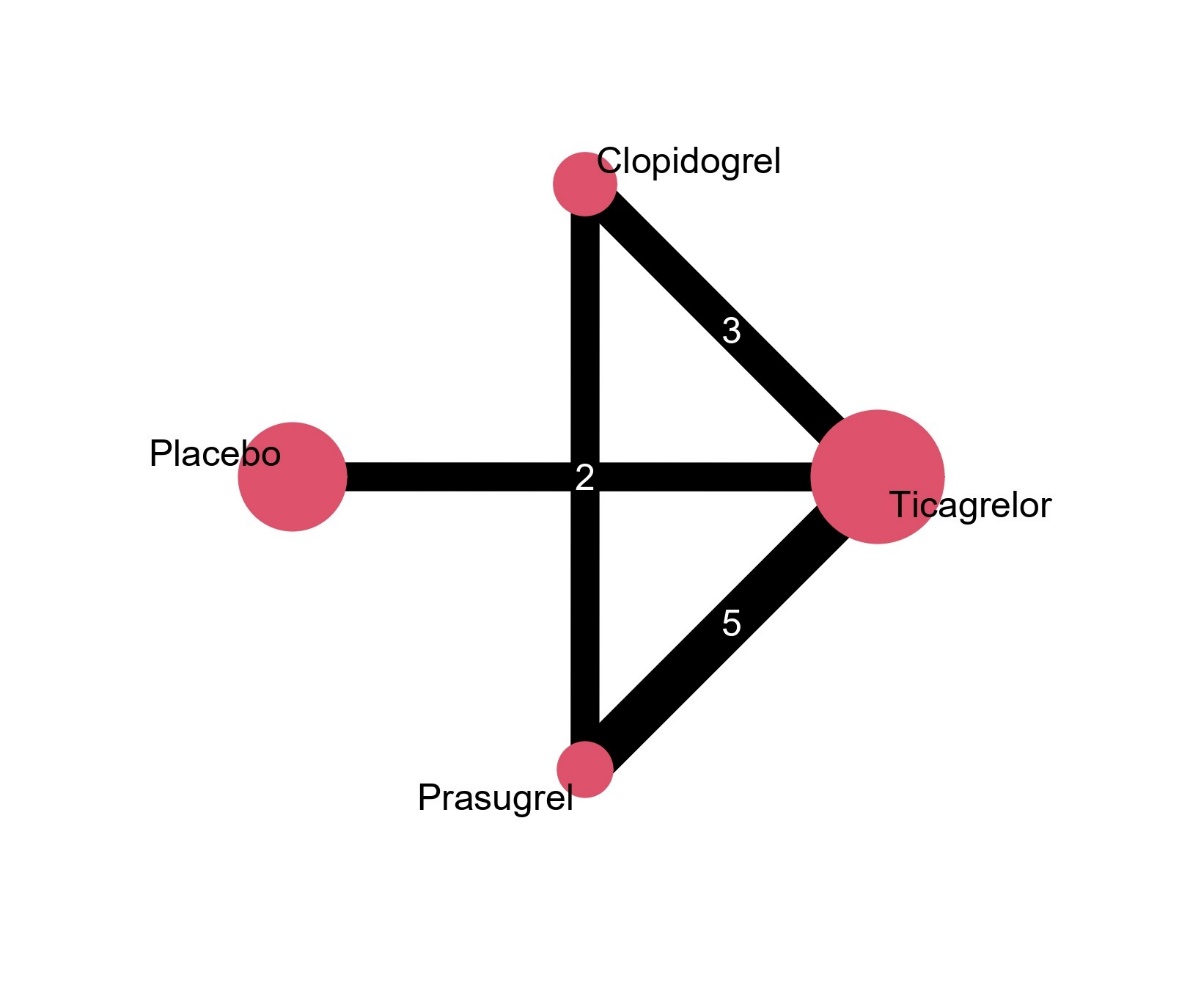


**Figure S5.** Funnel plot of cardiovascular mortality (CV mortality).


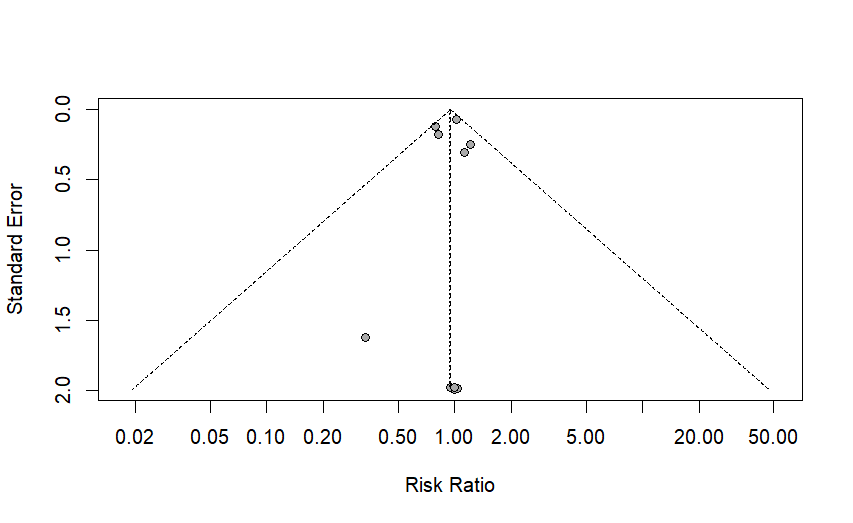


**Figure S6.** Network diagram of cardiovascular mortality.


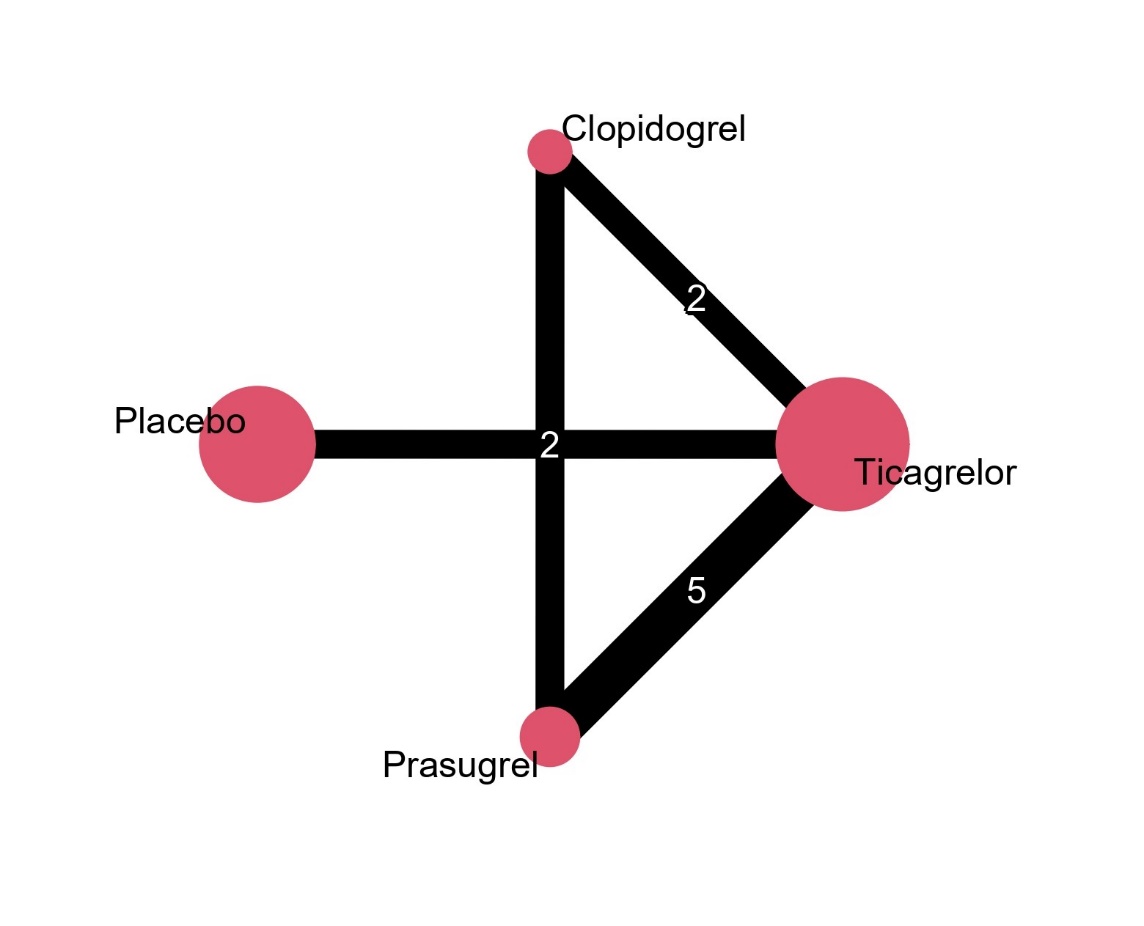


**Figure S7.** Funnel plot of all-cause mortality.


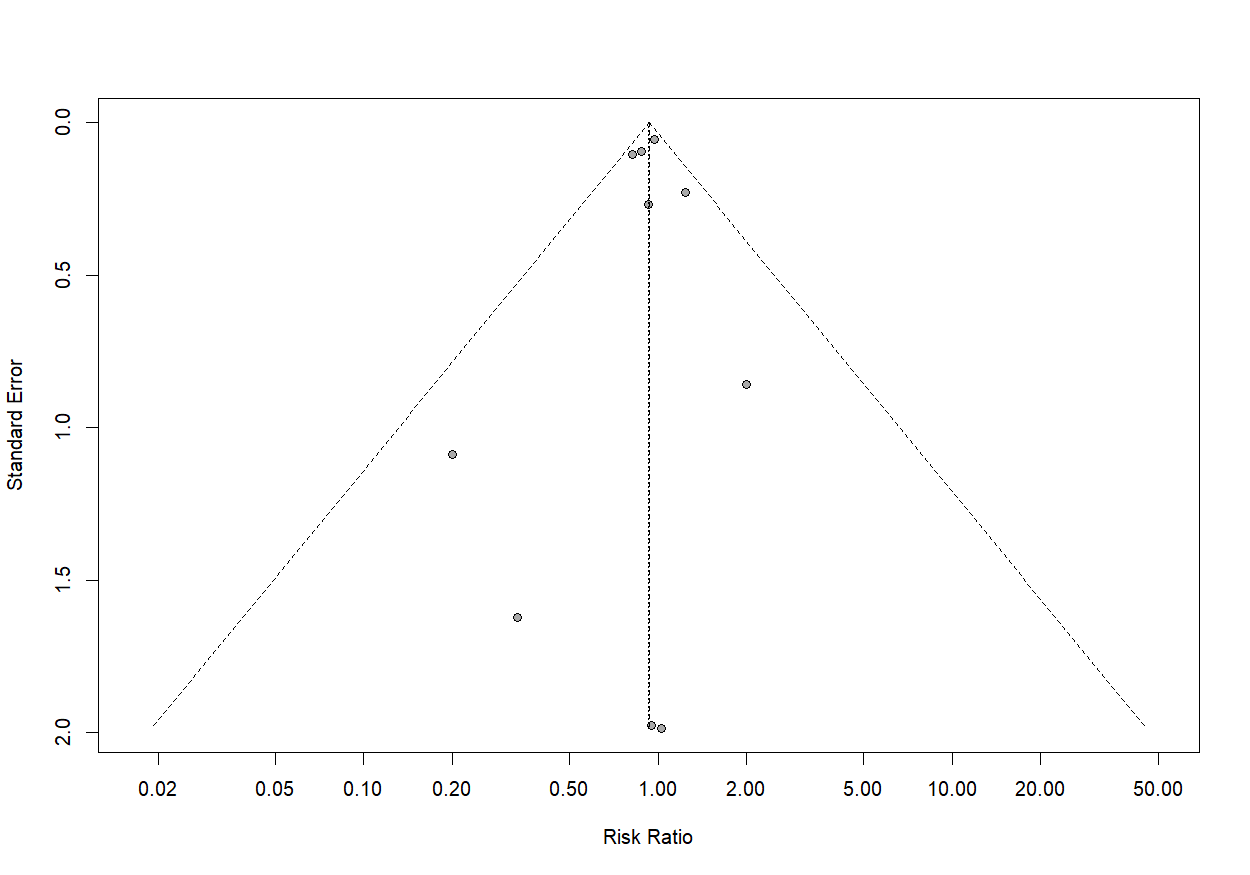


**Figure S8.** Network diagram of all-cause mortality.


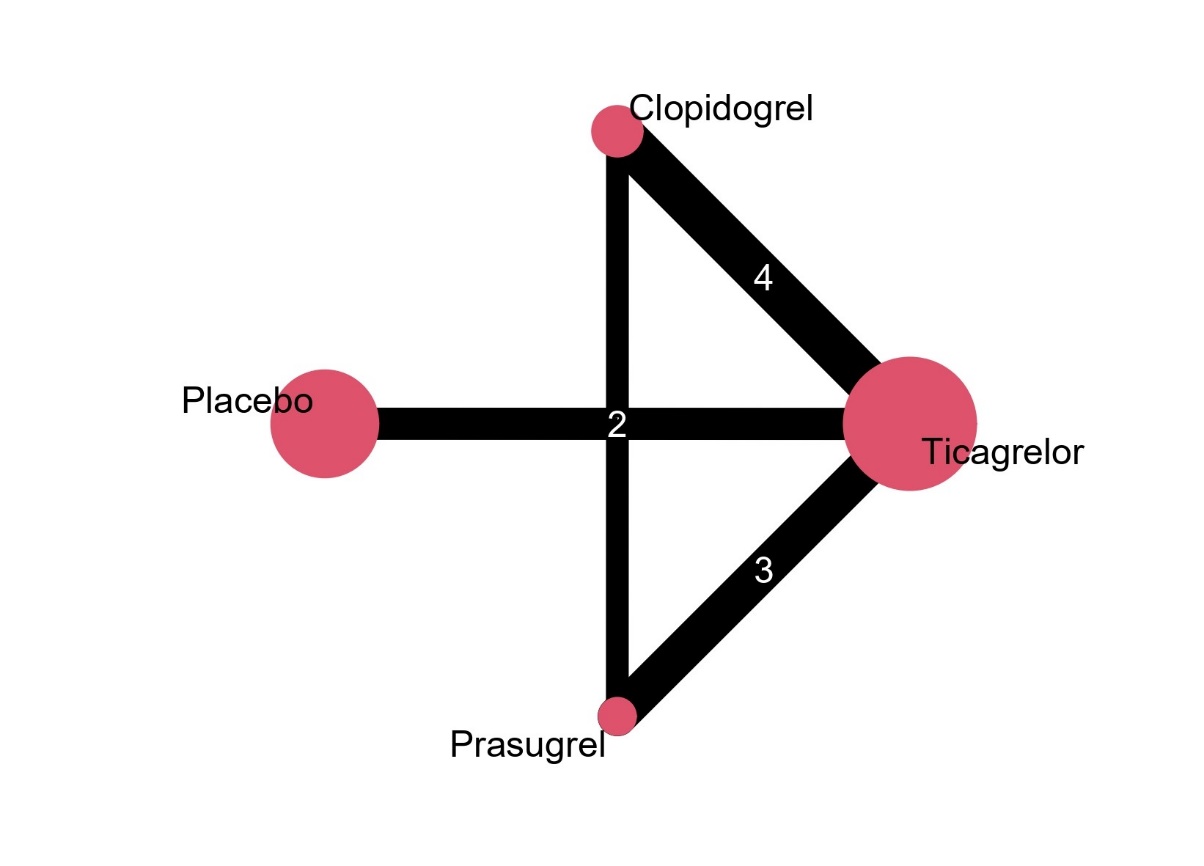


**Figure S9.** Network meta-analysis forest plot of myocardial infarction (MI).


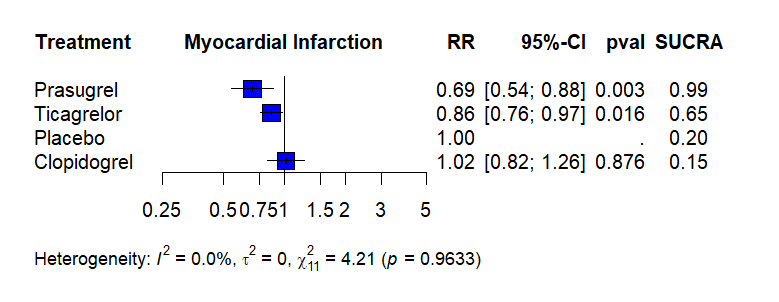


**Figure S10.** Pairwise meta-analysis forest plot of myocardial infarction (MI).


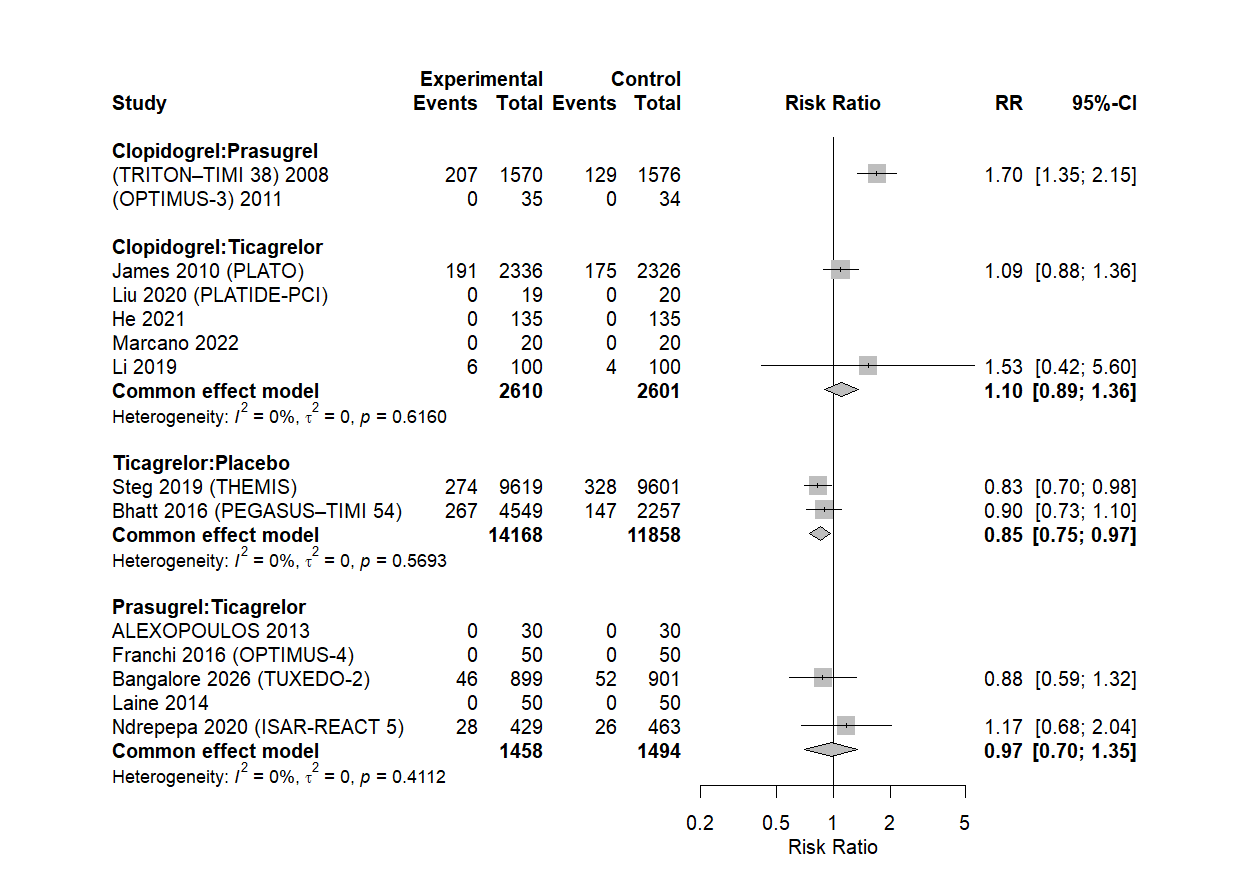


**Figure S11.** Funnel plot of myocardial infarction (MI).


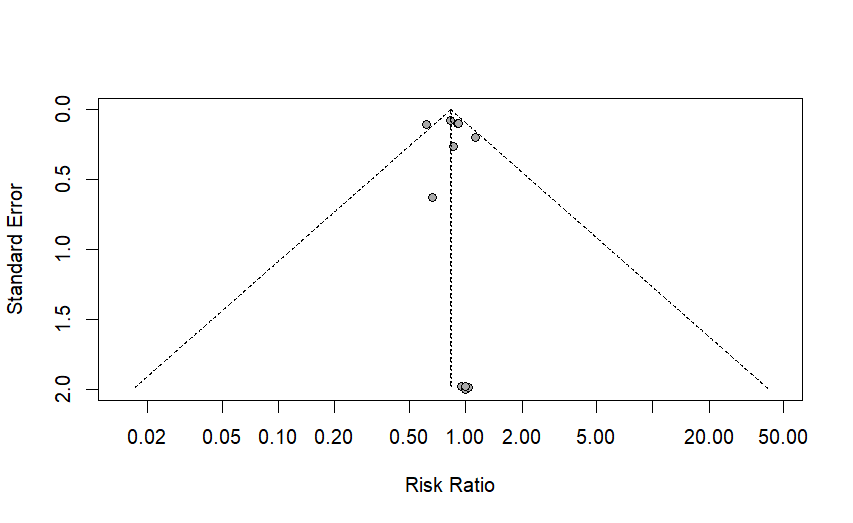


**Figure S12.** Network diagram of myocardial infarction (MI).


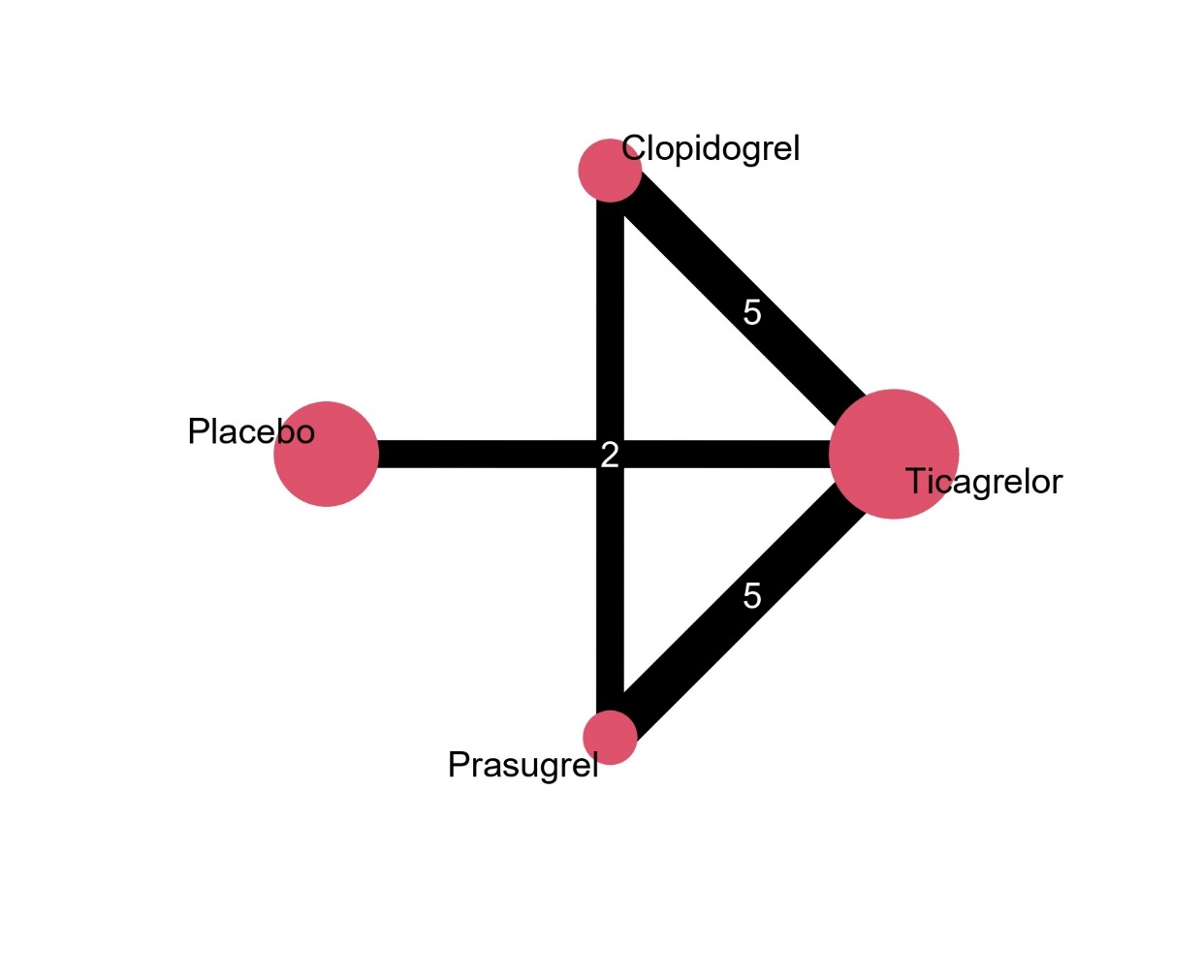


**Figure S13.** Network meta-analysis forest plot of stroke.


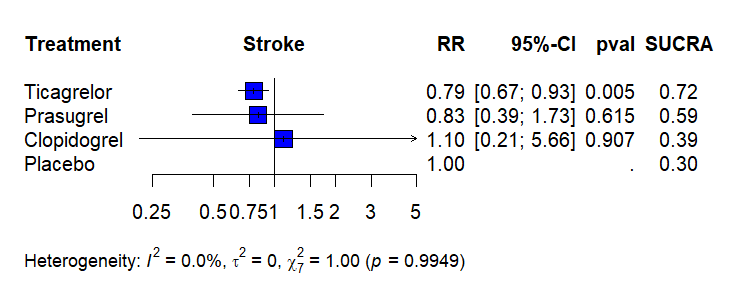


**Figure S14.** Pairwise meta-analysis forest plot of stroke.


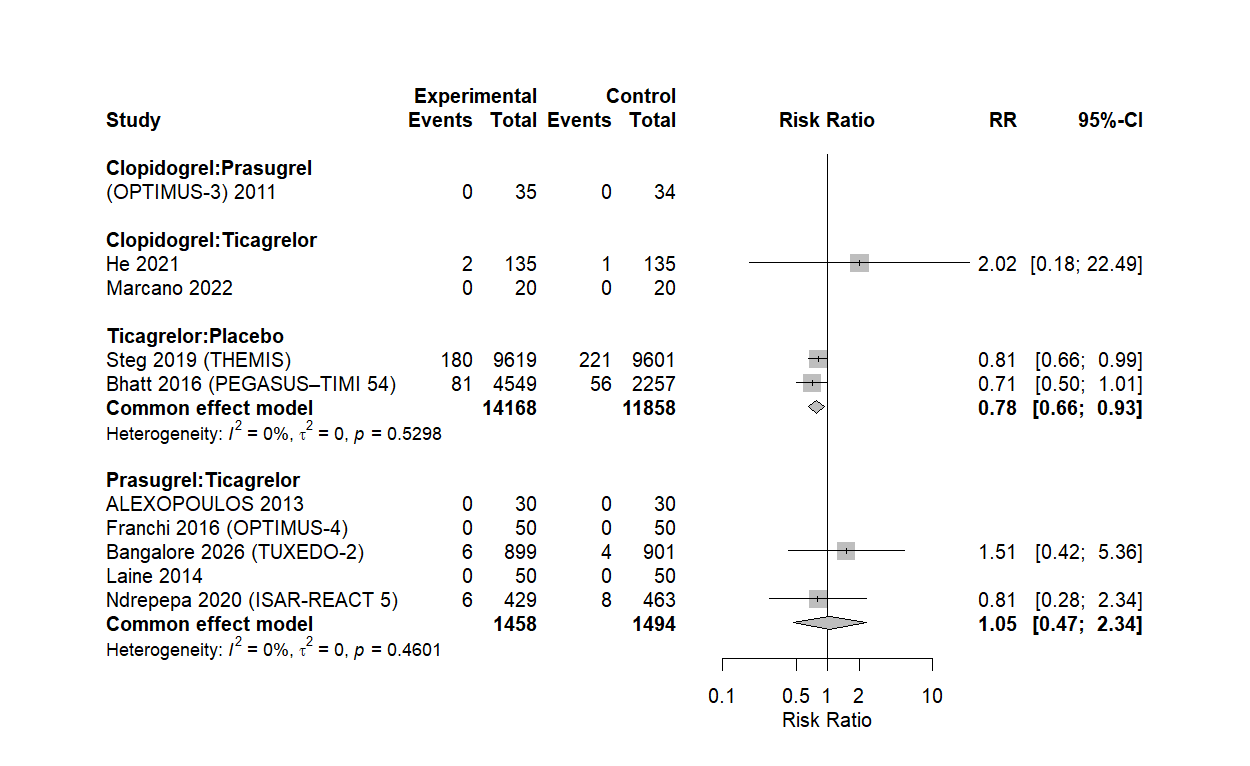


**Figure S15.** Funnel plot of stroke.


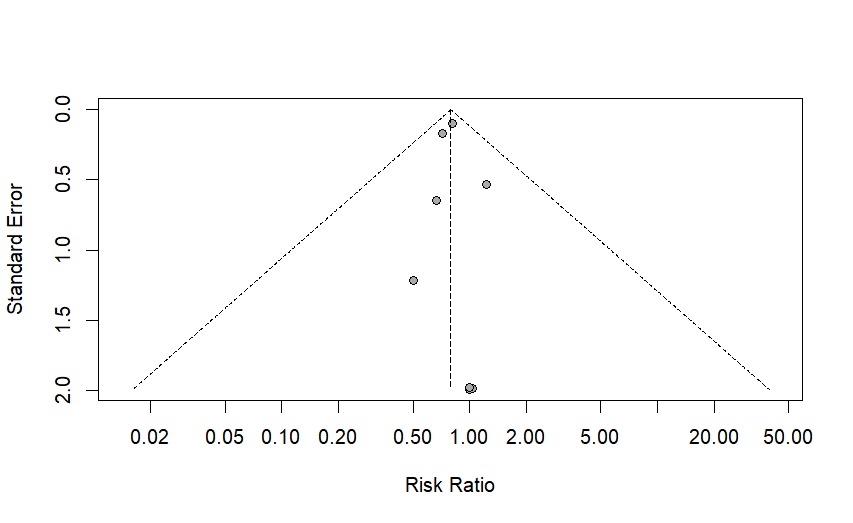


**Figure S16.** Network diagram of stroke.


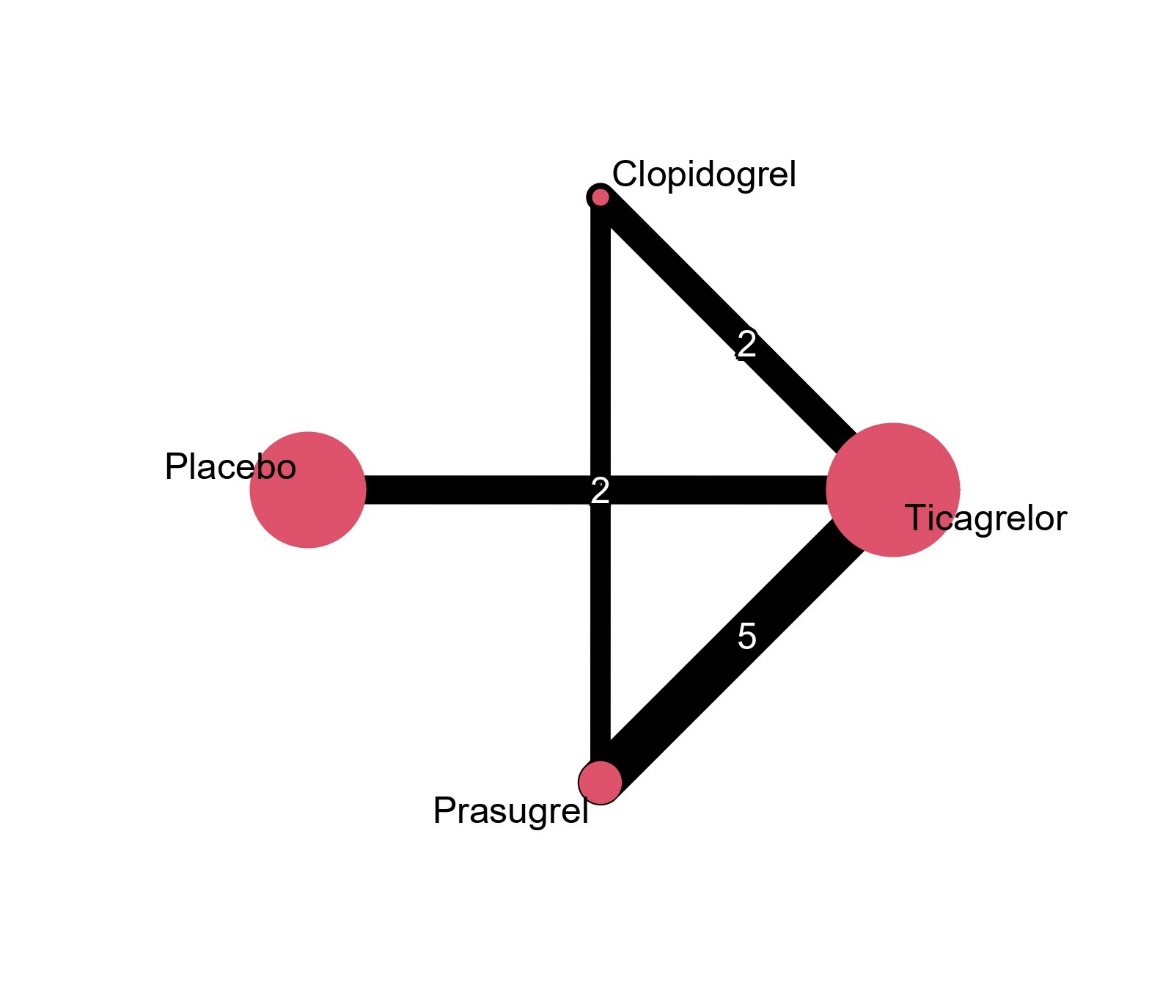


**Figure S17.** Network diagram of major bleeding (BARC 3–5).


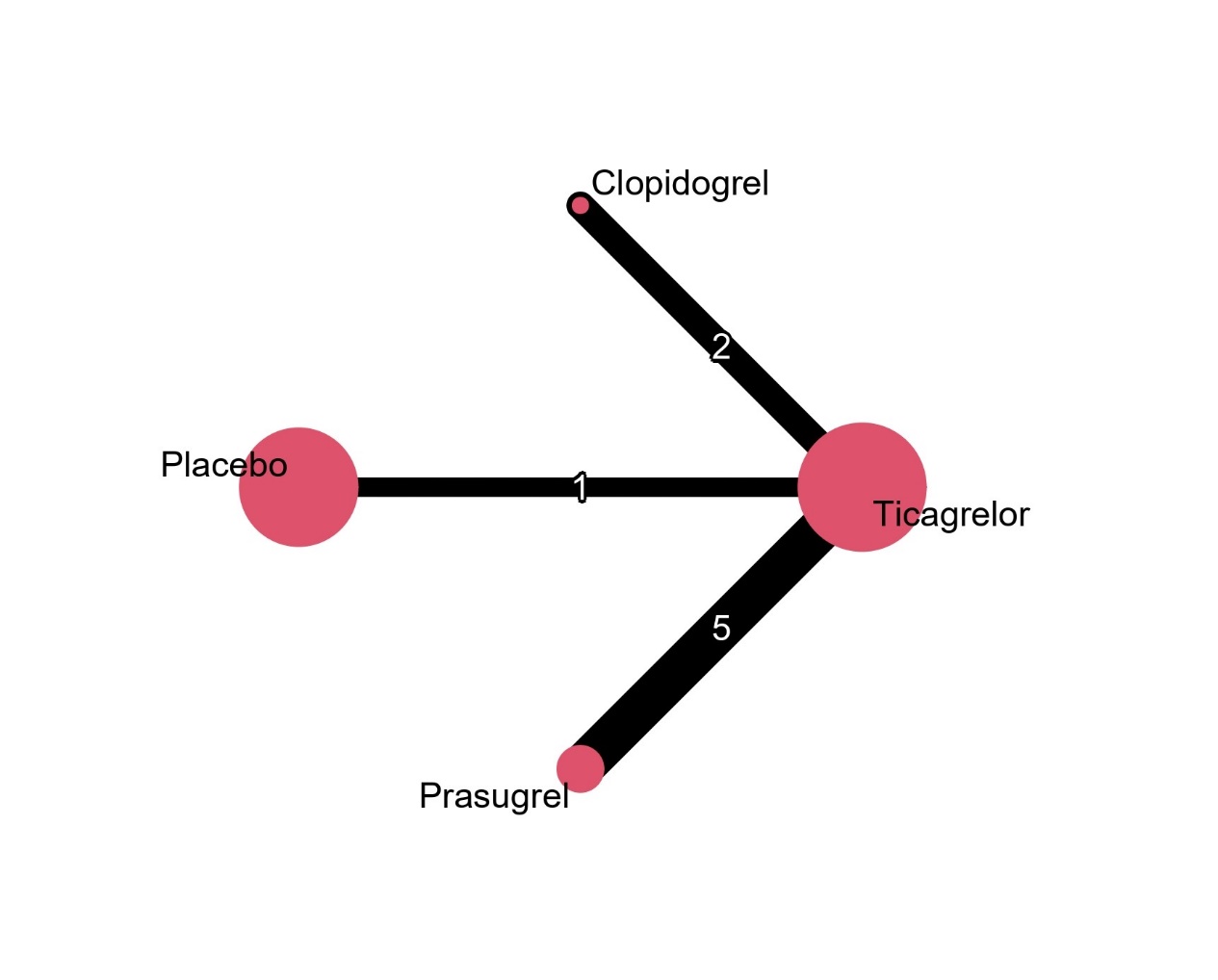


**Figure S18.** Network meta-analysis forest plot of Thrombolysis in Myocardial Infarction major bleeding (TIMI major bleeding).


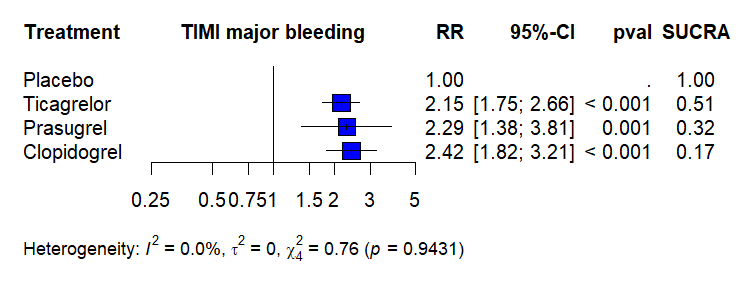


**Figure S19.** Pairwise meta-analysis forest plot of Thrombolysis in Myocardial Infarction major bleeding (TIMI major bleeding).


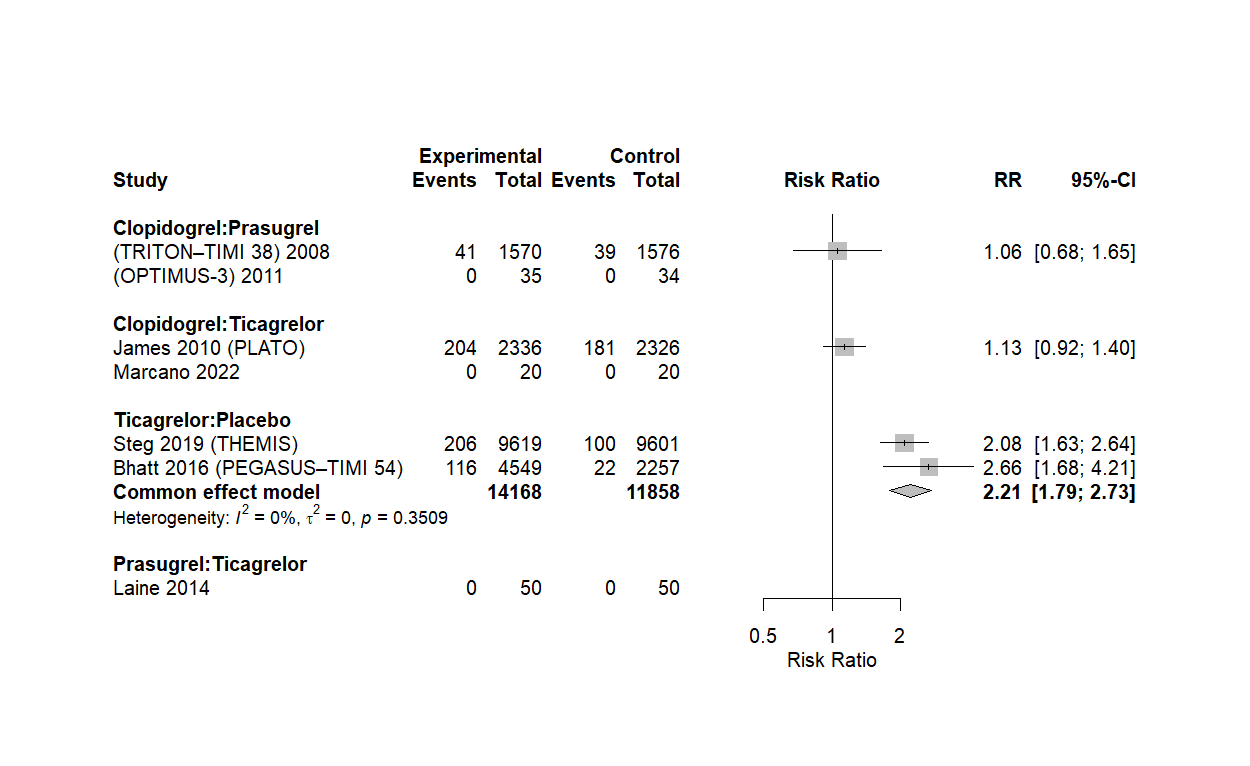


**Figure S20.** Network diagram of Thrombolysis in Myocardial Infarction major bleeding (TIMI major bleeding).


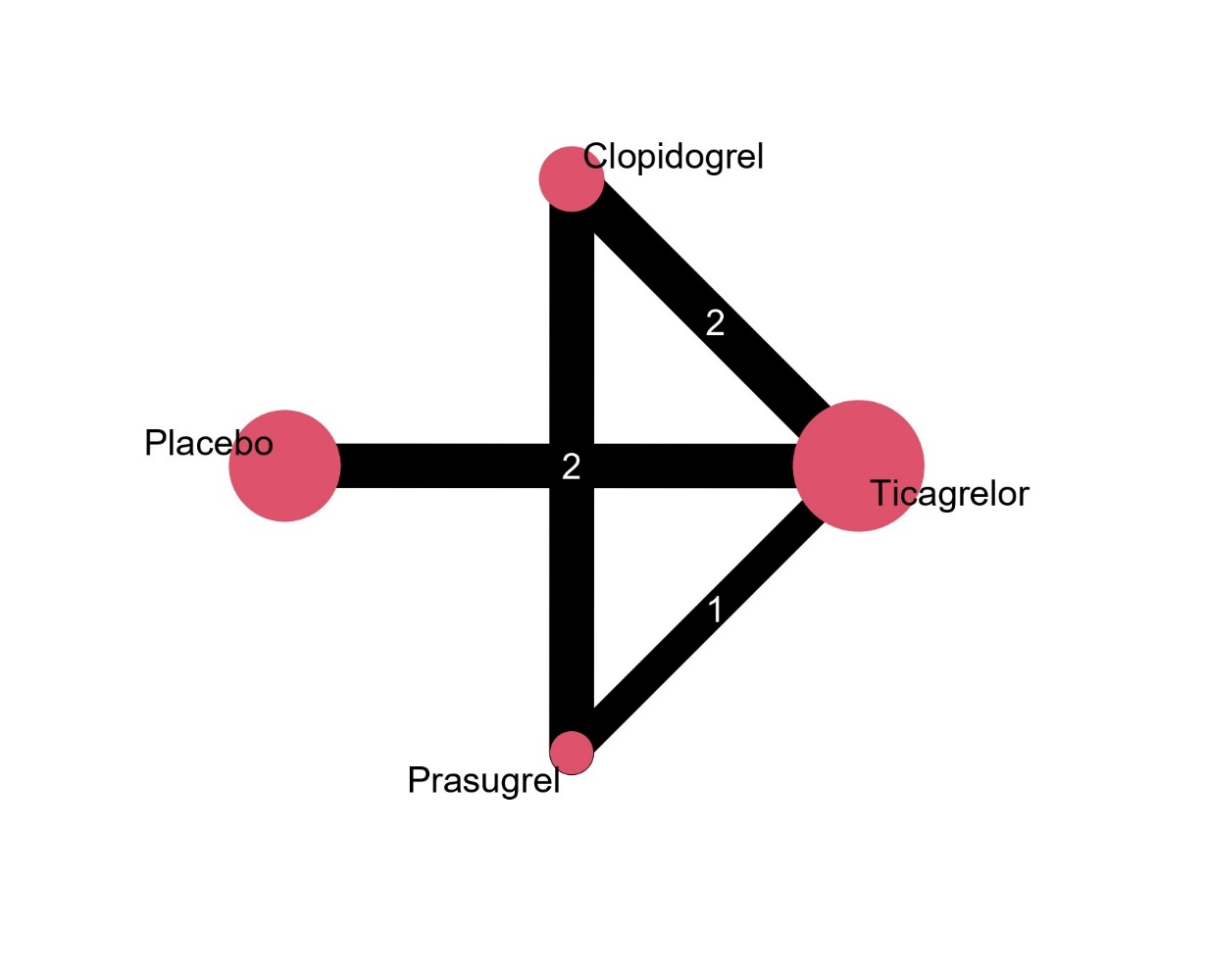


**Figure S21.** Network meta-analysis forest plot of Platelet Inhibition and Patient Outcomes major bleeding (PLATO major bleeding).


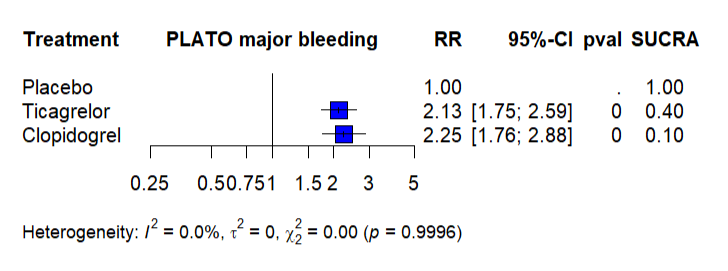


**Figure S22.** Pairwise meta-analysis forest plot of Platelet Inhibition and Patient Outcomes major bleeding (PLATO major bleeding).


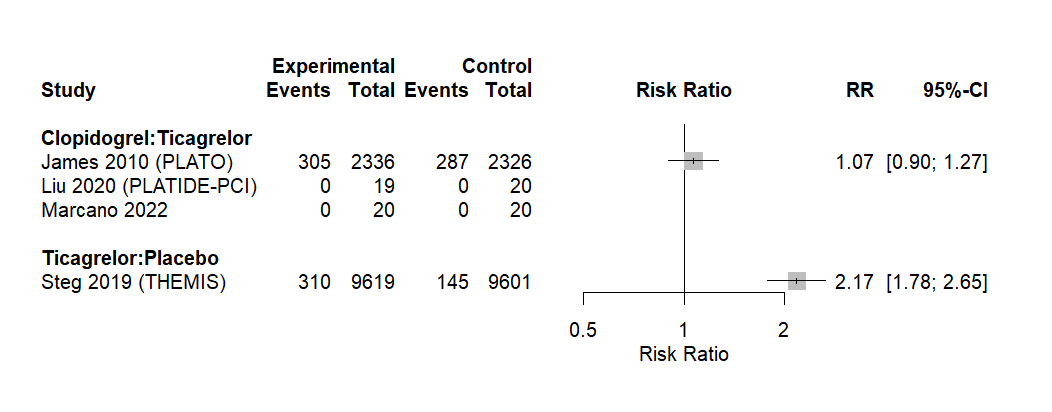


**Figure S23.** Network diagram of Platelet Inhibition and Patient Outcomes major bleeding (PLATO major bleeding).


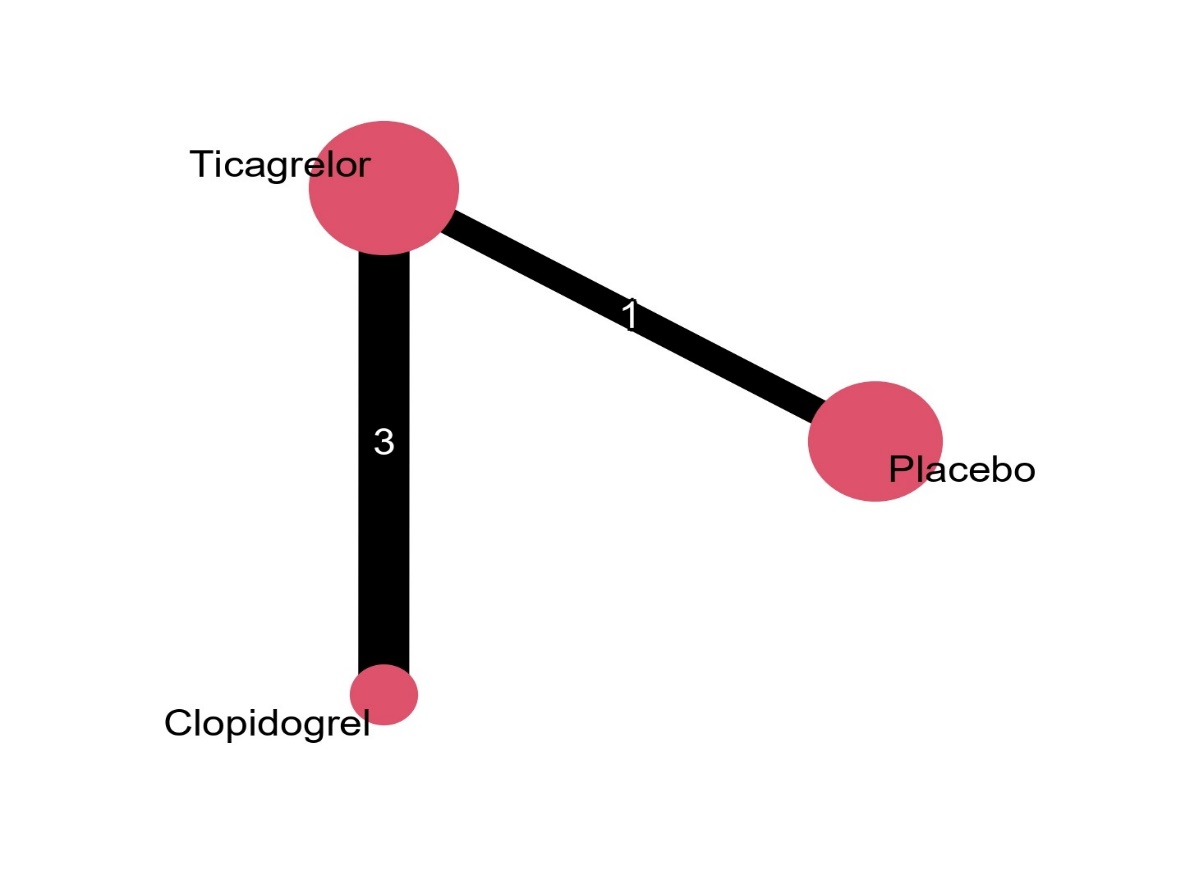


**Figure S24.** Network meta-analysis forest plot of dyspnea.


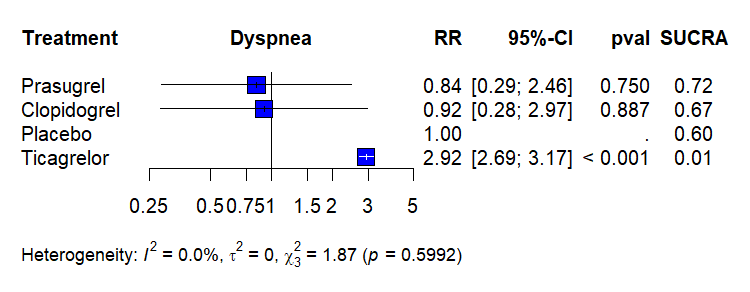


**Figure S25.** Pairwise meta-analysis forest plot of dyspnea.


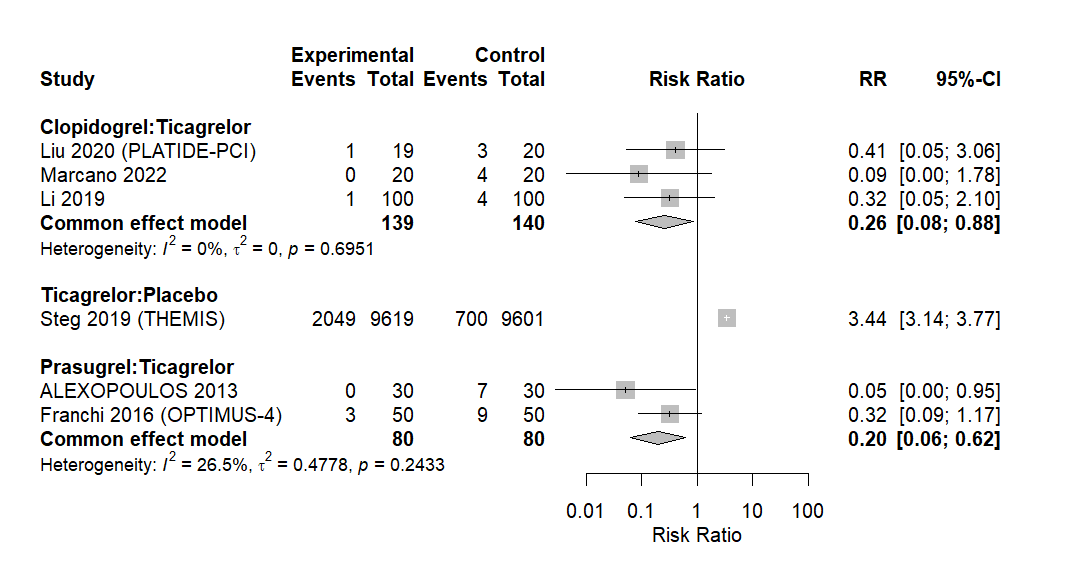


**Figure S26.** Network diagram of dyspnea.


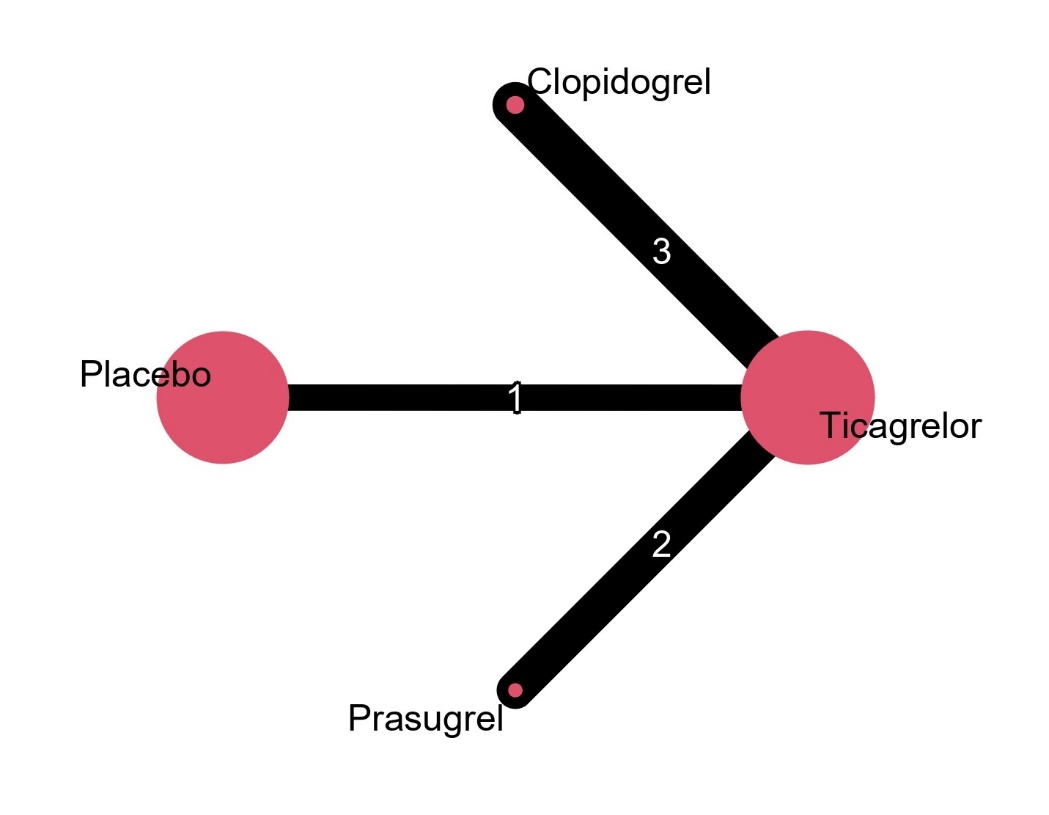


**Figure S27.** Network meta-analysis forest plot of platelet reactivity (platelet reactivity units, PRU).


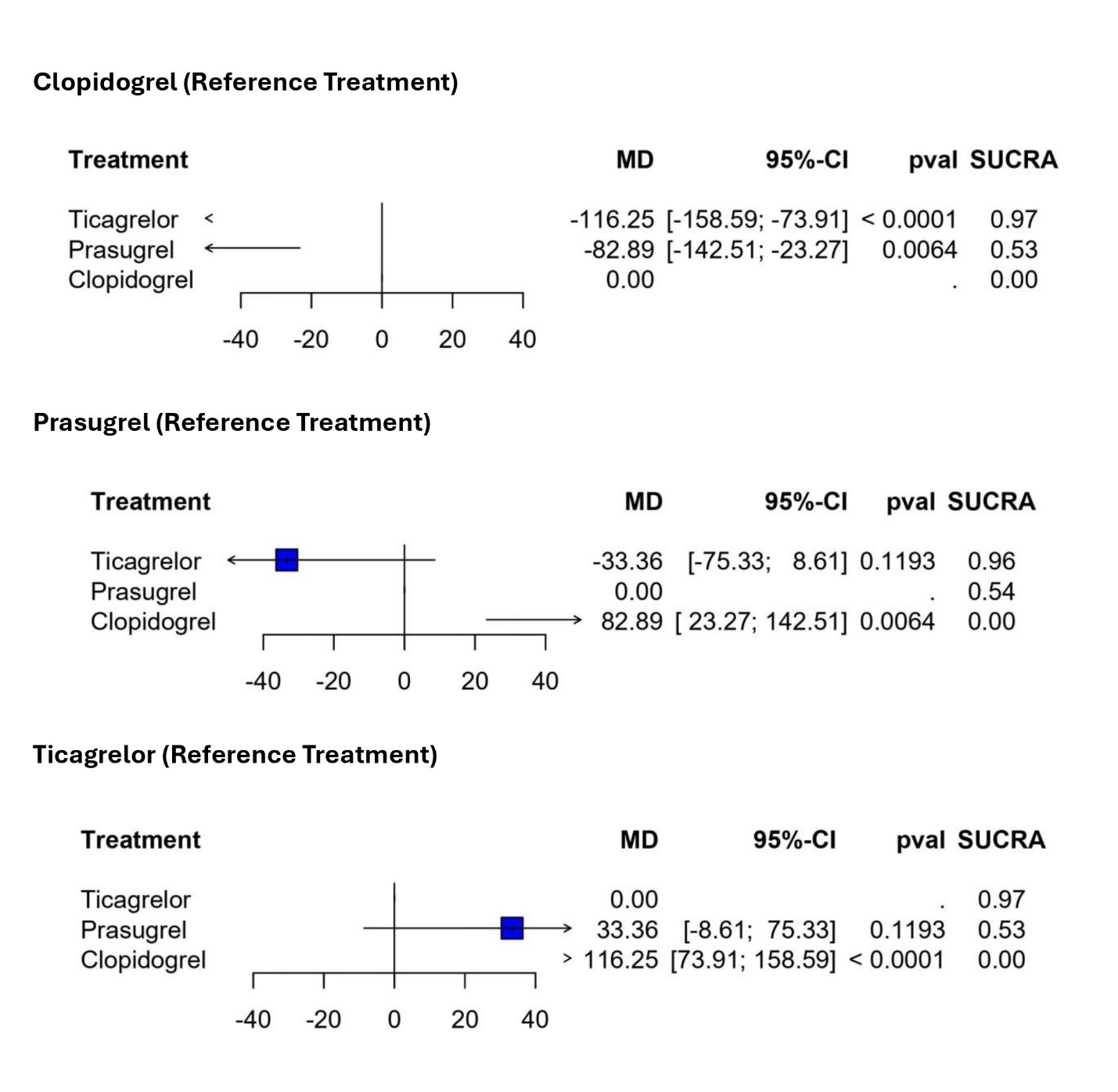


**Figure S28.** Pairwise meta-analysis forest plot of platelet reactivity (platelet reactivity units, PRU).


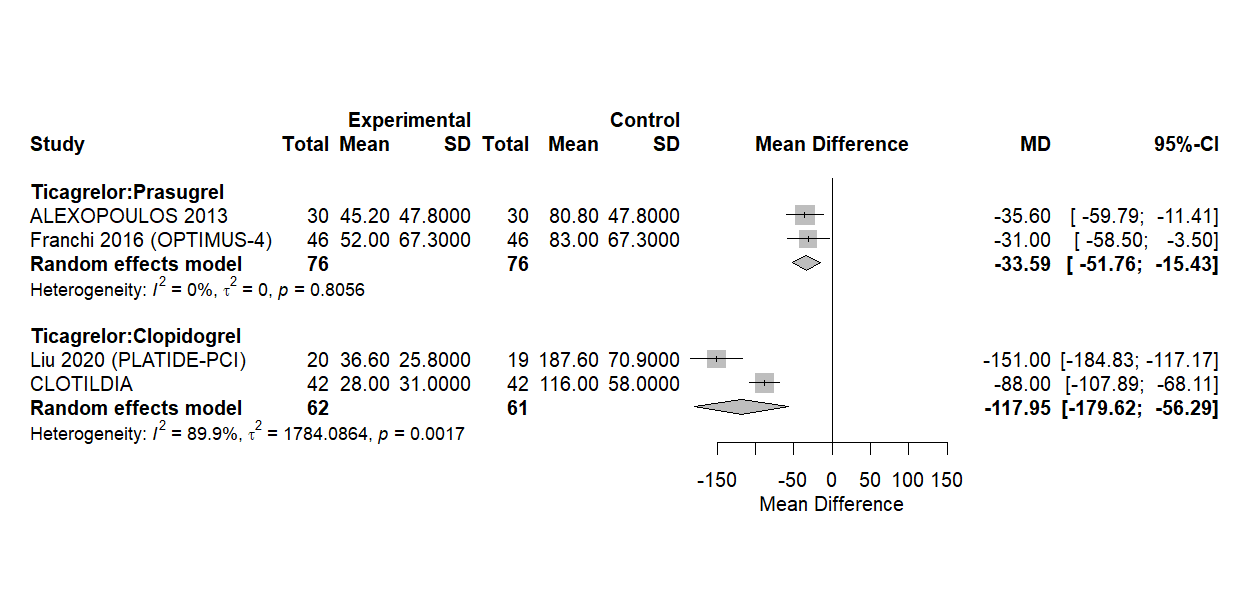


**Figure S29.** Network diagram of platelet reactivity (platelet reactivity units, PRU).


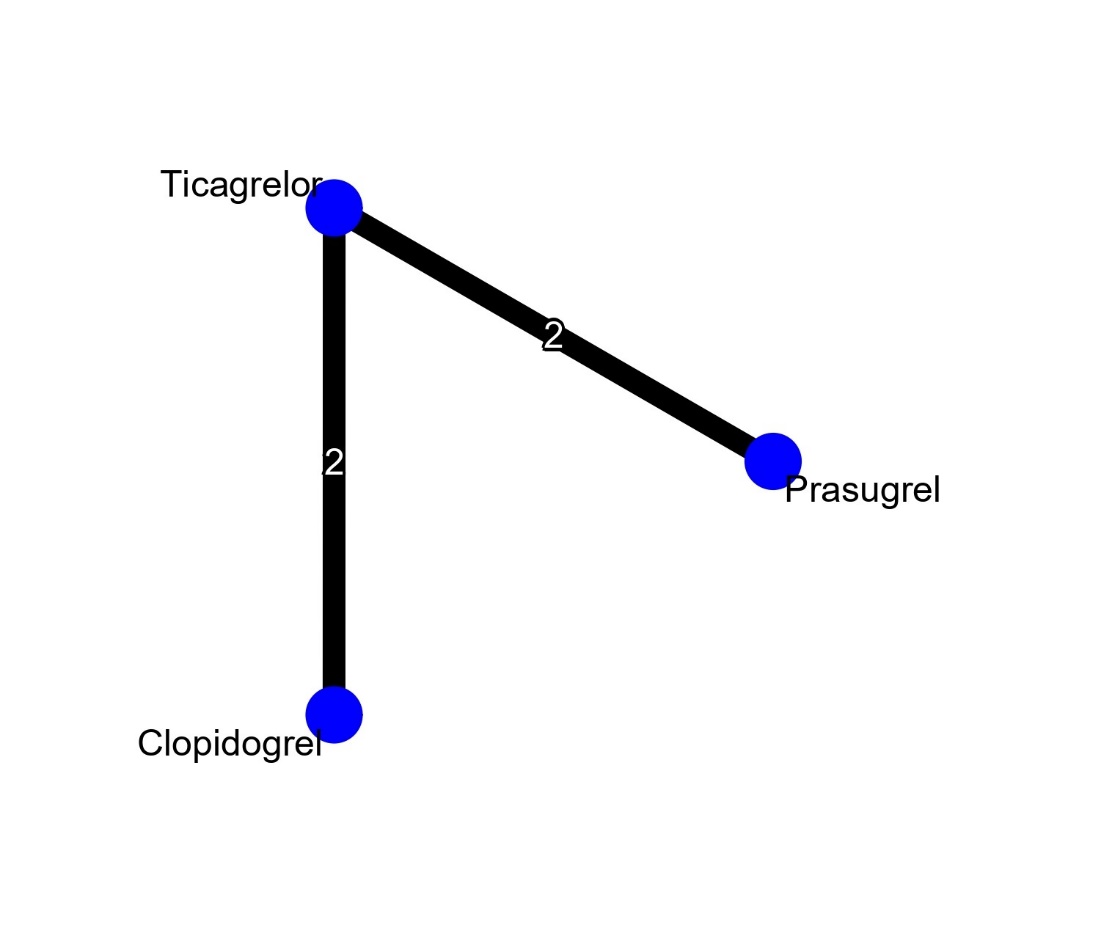


**Figure S30.** Network meta-analysis forest plot of percent platelet inhibition.


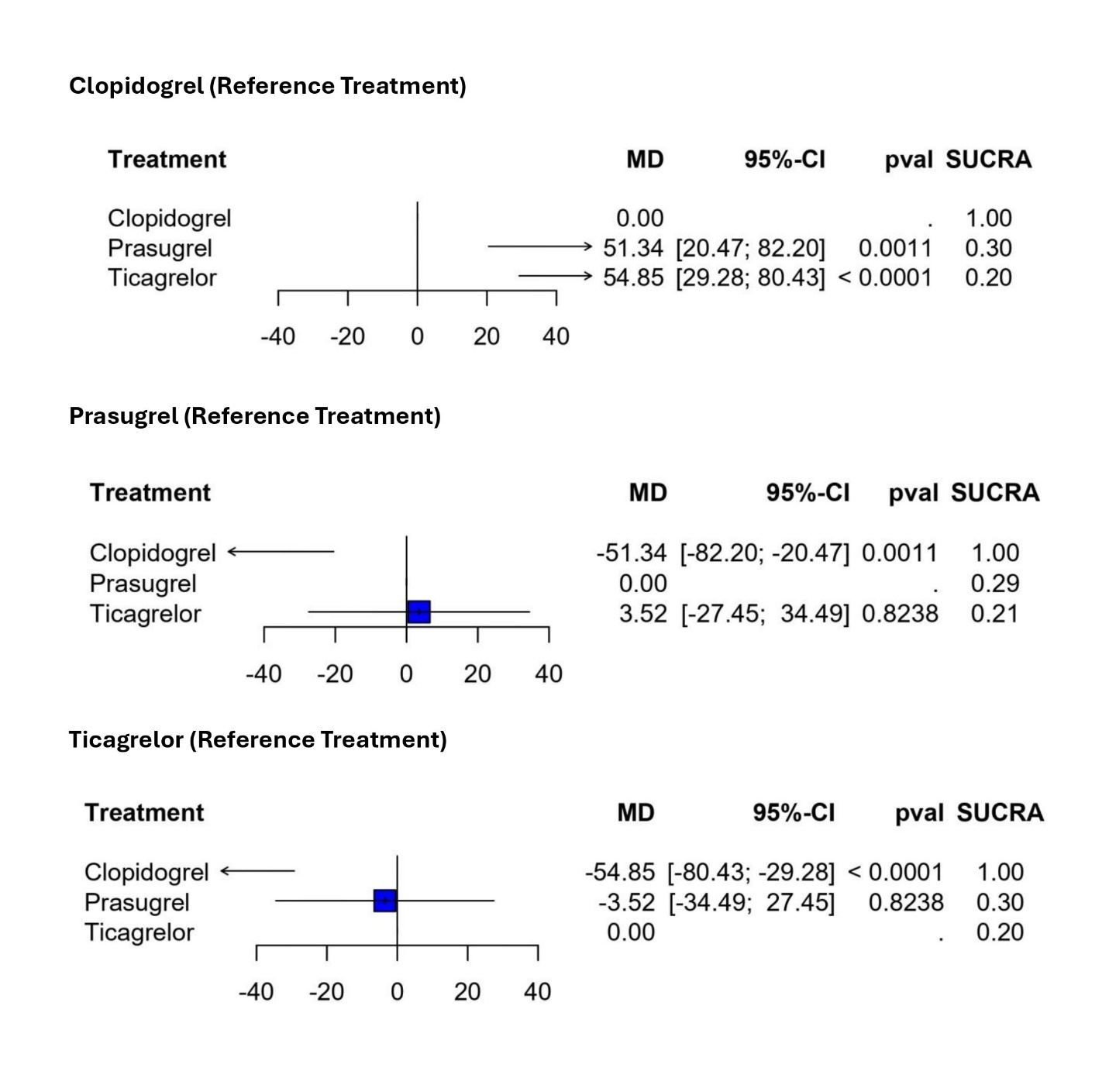


**Figure S31.** Pairwise meta-analysis forest plot of percent platelet inhibition.


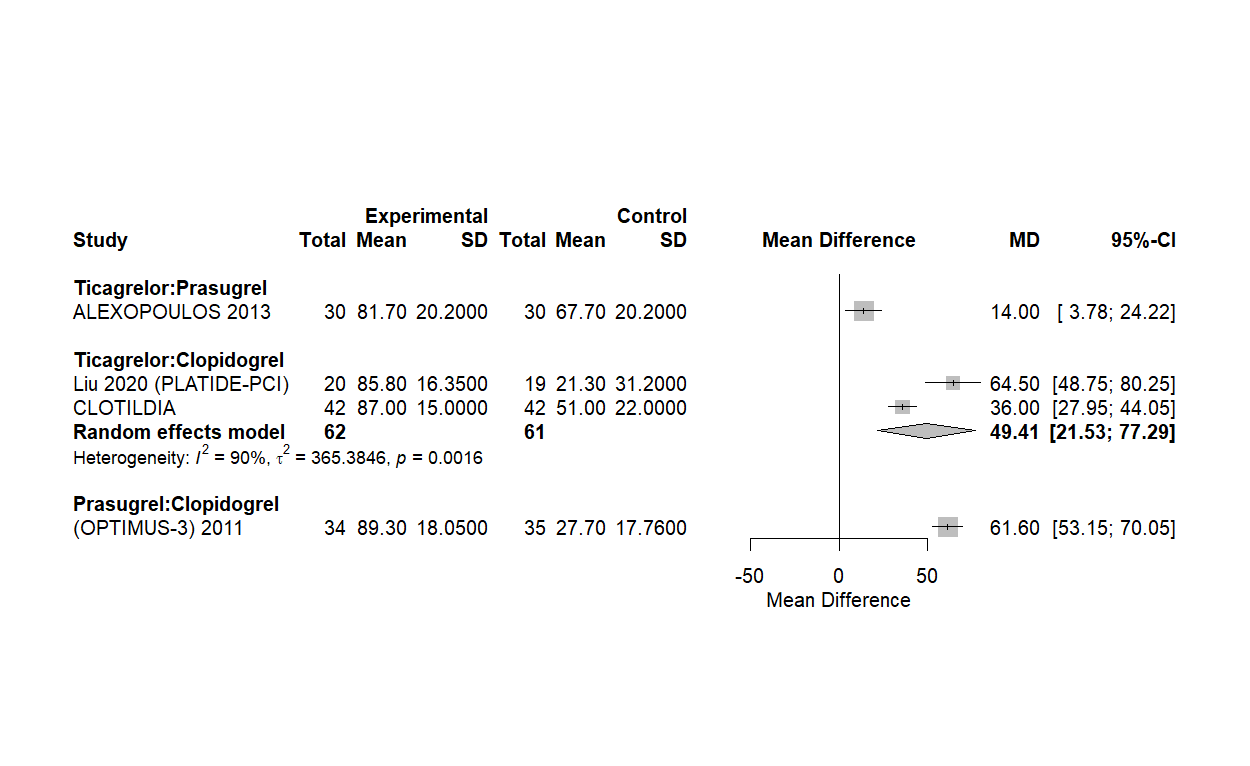


**Figure S32.** Network diagram of percent platelet inhibition
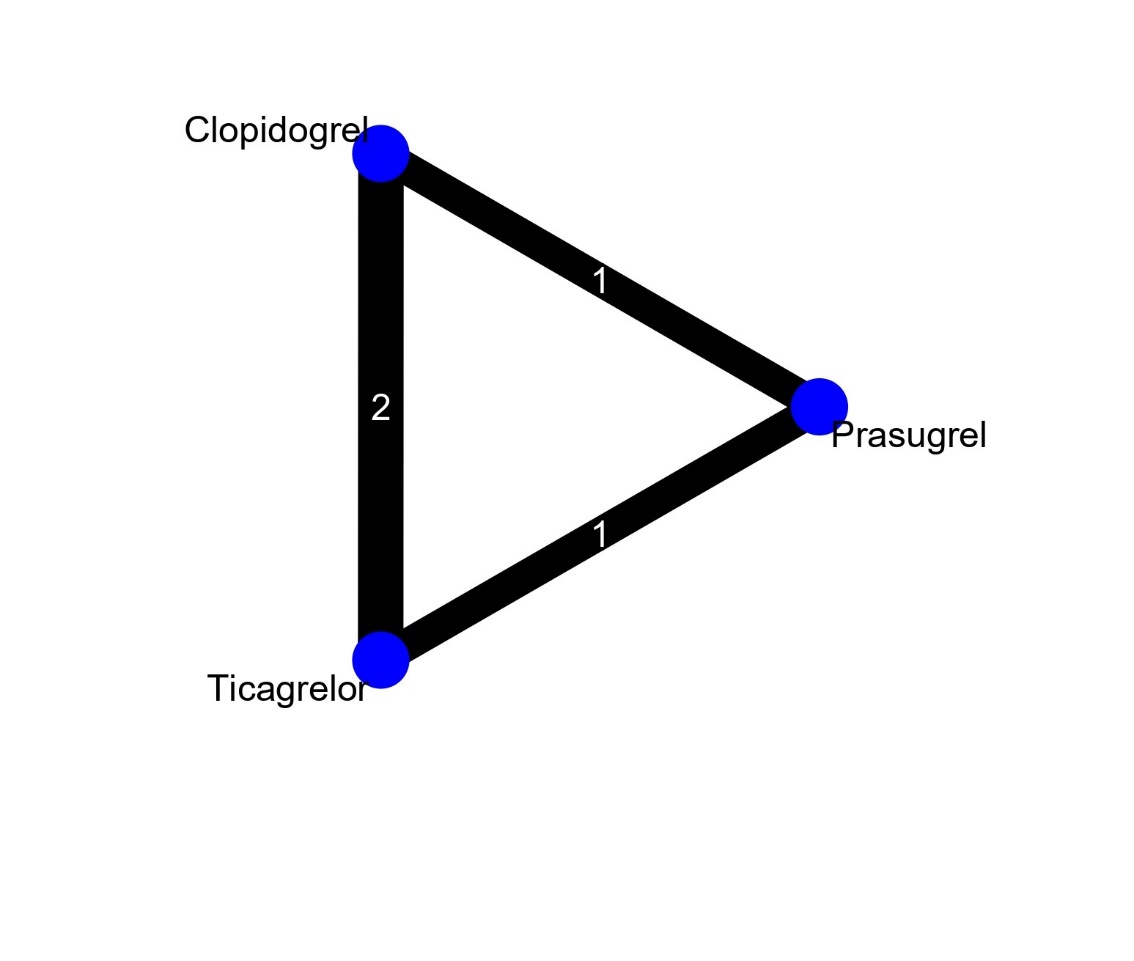


**Figure S33.** Sensitivity network meta-analysis for MACE after exclusion of pharmacodynamic-only studies.


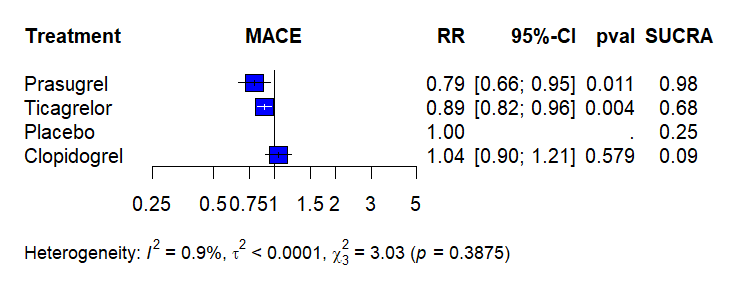


**Figure S34.** Sensitivity network meta-analysis for myocardial infarction after exclusion of pharmacodynamic-only studies.


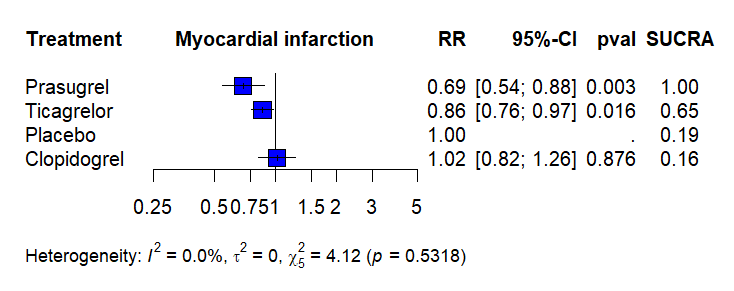


**Figure S35.** Sensitivity network meta-analysis for stroke after exclusion of pharmacodynamic-only studies.


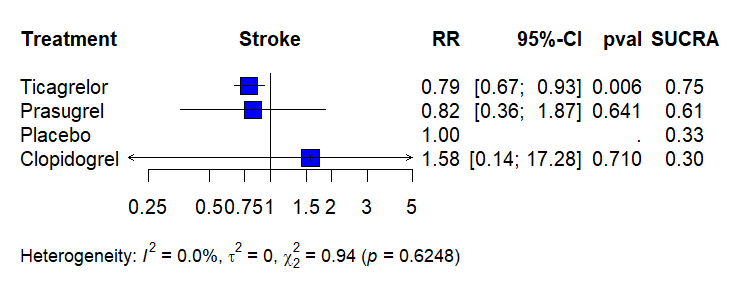


**Figure S36.** Sensitivity network meta-analysis for cardiovascular mortality after exclusion of pharmacodynamic-only studies.


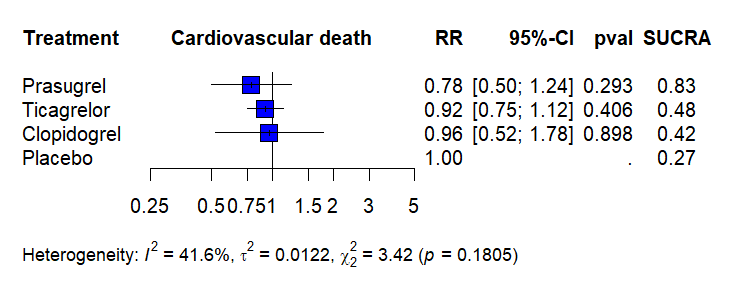


**Figure S37.** Sensitivity network meta-analysis for all-cause mortality after exclusion of pharmacodynamic-only studies.


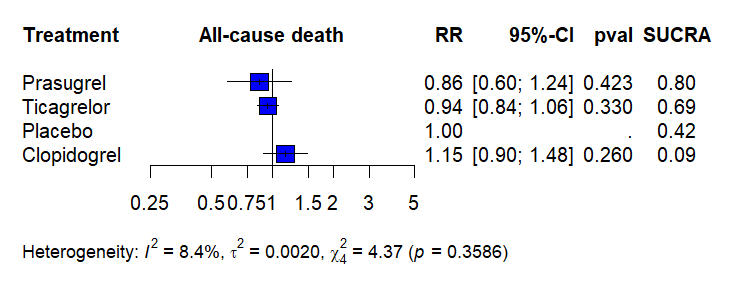


**Figure S38.** Sensitivity network meta-analysis for BARC 3–5 major bleeding after exclusion of pharmacodynamic-only studies.


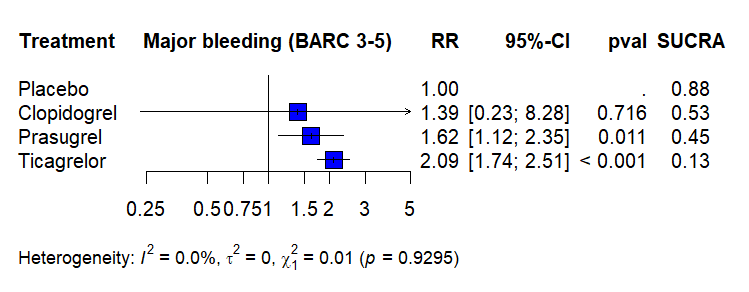


**Figure S39.** Sensitivity network meta-analysis for TIMI major bleeding after exclusion of pharmacodynamic-only studies.


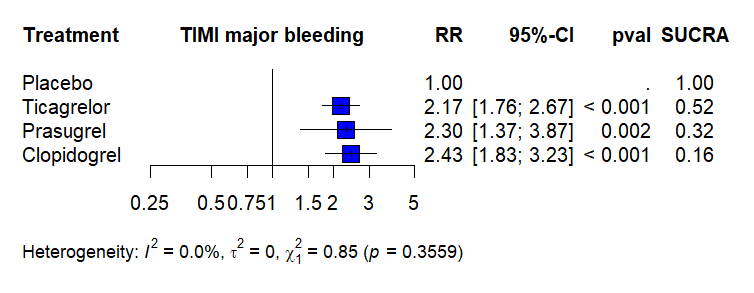


**Figure S40.** Sensitivity network meta-analysis for PLATO major bleeding after exclusion of pharmacodynamic-only studies.


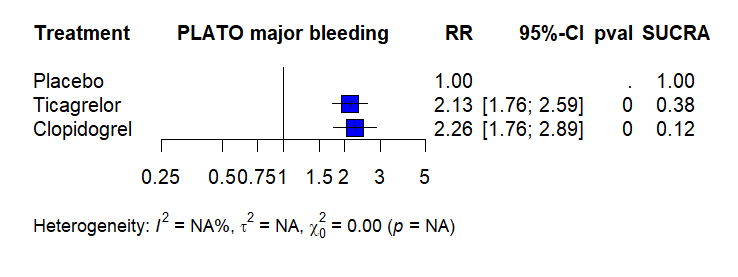


**Figure S41.** Sensitivity network meta-analysis for dyspnea after exclusion of pharmacodynamic-only studies.


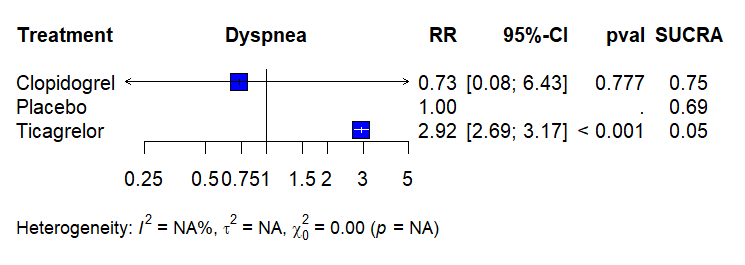


**Figure S42.** Ranking of treatment strategies based on P-scores across efficacy outcomes.


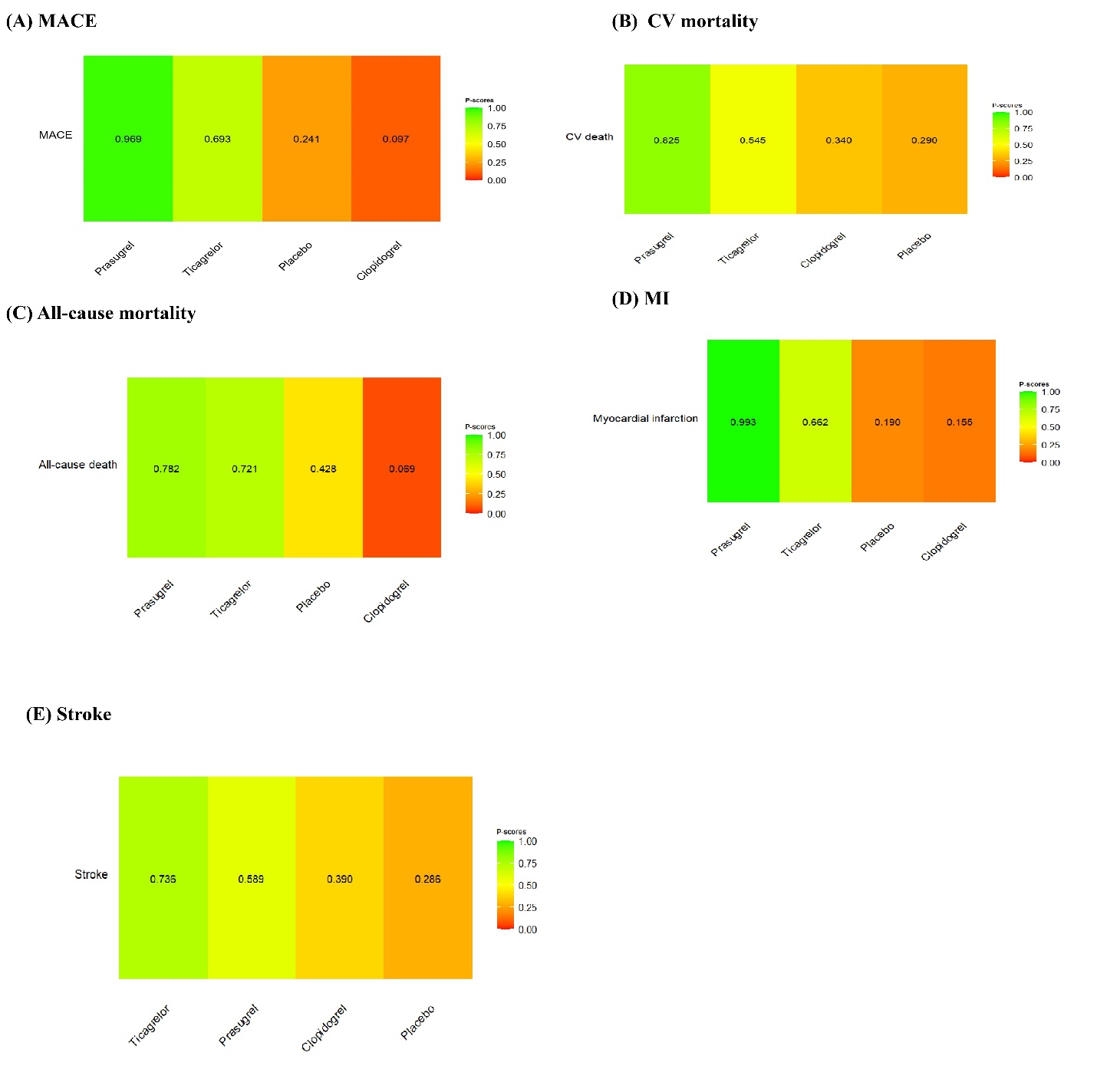


**Figure S43.** Ranking of treatment strategies based on P-scores across safety outcomes.


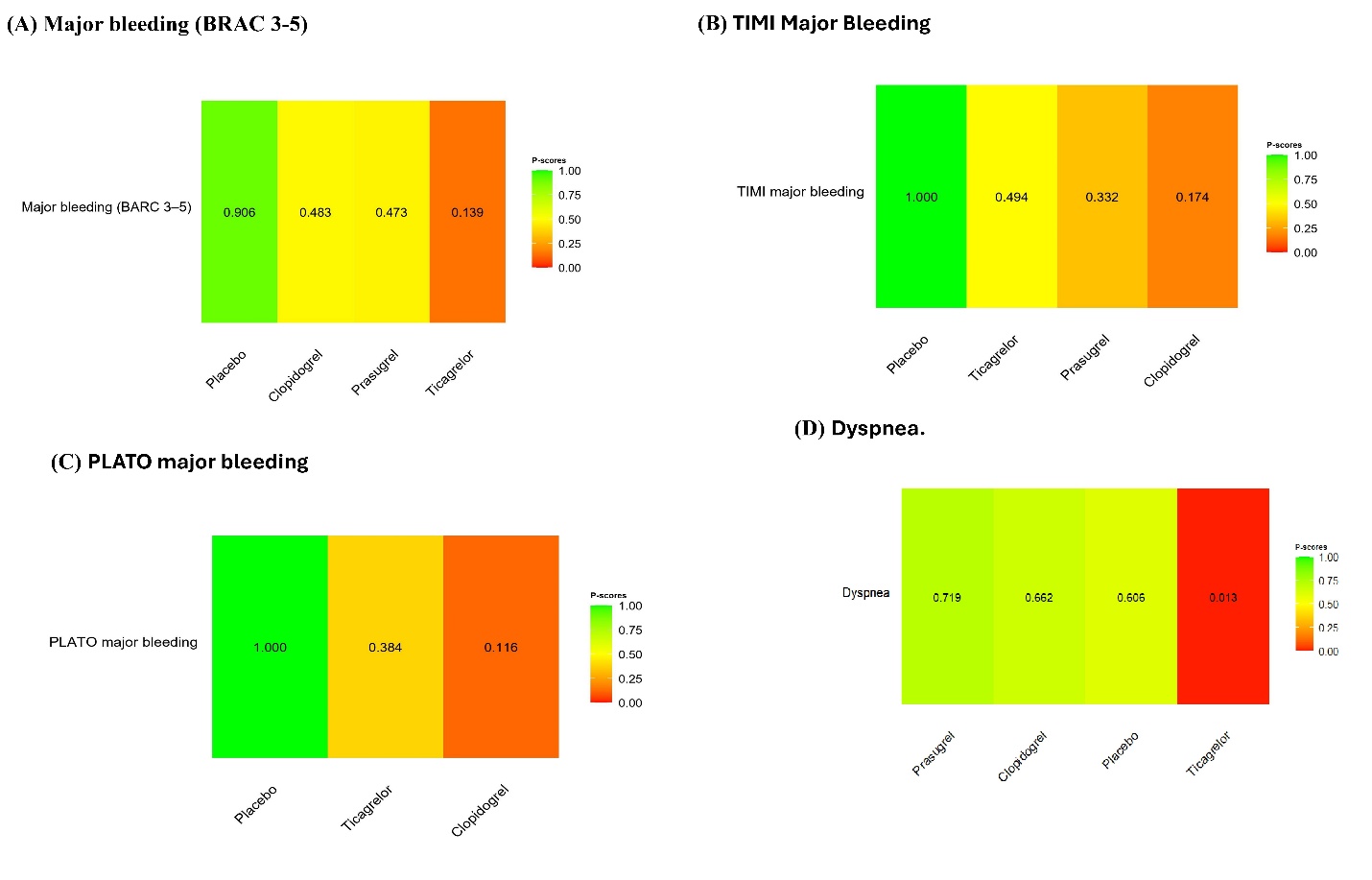


**Supplementary Table 5.** Assessment of Consistency of the Network Meta-Analysis Model

| Outcome | Comparison | k | Prop | NMA | Direct | Indirect | RoR | Z | P-value |
| --- | --- | --- | --- | --- | --- | --- | --- | --- | --- |
| MACE | Clopidogrel vs Prasugrel | 2 | 0.71 | 1.31 | 1.39 | 1.13 | 1.23 | 1.28 | 0.201 |
|  | Clopidogrel vs Ticagrelor | 3 | 0.8 | 1.17 | 1.13 | 1.39 | 0.81 | -1.28 | 0.201 |
|  | Prasugrel vs Ticagrelor | 5 | 0.49 | 0.9 | 1 | 0.81 | 1.23 | 1.28 | 0.201 |
| CV death | Clopidogrel vs Prasugrel | 2 | 0.98 | 1.22 | 1.22 | 1.18 | 1.04 | 0.03 | 0.978 |
|  | Clopidogrel vs Ticagrelor | 2 | 0.03 | 1.06 | 1.03 | 1.07 | 0.96 | -0.03 | 0.978 |
|  | Prasugrel vs Ticagrelor | 5 | 0.98 | 0.87 | 0.87 | 0.84 | 1.04 | 0.03 | 0.978 |
| All-cause death | Clopidogrel vs Prasugrel | 1 | 0.01 | 1.32 | 0.97 | 1.32 | 0.74 | -0.15 | 0.878 |
|  | Clopidogrel vs Ticagrelor | 4 | 1 | 1.22 | 1.22 | 0.9 | 1.36 | 0.15 | 0.878 |
|  | Prasugrel vs Ticagrelor | 3 | 0.99 | 0.93 | 0.93 | 1.26 | 0.74 | -0.15 | 0.878 |
| Myocardial infarction | Clopidogrel vs Prasugrel | 2 | 0.75 | 1.47 | 1.61 | 1.12 | 1.44 | 1.7 | 0.089 |
|  | Clopidogrel vs Ticagrelor | 5 | 0.78 | 1.18 | 1.09 | 1.57 | 0.7 | -1.7 | 0.089 |
|  | Prasugrel vs Ticagrelor | 5 | 0.46 | 0.81 | 0.98 | 0.68 | 1.44 | 1.7 | 0.089 |
| Stroke | Clopidogrel vs Prasugrel | 1 | 0.2 | 1.33 | 0.97 | 1.44 | 0.67 | -0.18 | 0.859 |
|  | Clopidogrel vs Ticagrelor | 2 | 0.83 | 1.4 | 1.5 | 1.01 | 1.48 | 0.18 | 0.859 |
|  | Prasugrel vs Ticagrelor | 5 | 0.97 | 1.05 | 1.04 | 1.54 | 0.67 | -0.18 | 0.859 |
| TIMI major bleeding | Clopidogrel vs Prasugrel | 2 | 0.99 | 1.05 | 1.05 | 1.12 | 0.94 | -0.03 | 0.975 |
|  | Clopidogrel vs Ticagrelor | 2 | 1 | 1.12 | 1.12 | 1.05 | 1.06 | 0.03 | 0.975 |
|  | Prasugrel vs Ticagrelor | 1 | 0.01 | 1.06 | 1 | 1.06 | 0.94 | -0.03 | 0.975 |

**Abbreviations:** k = number of studies providing direct evidence; Prop = proportion of direct evidence; NMA = estimated treatment effect from network meta-analysis; Direct = estimated treatment effect from direct evidence; Indirect = estimated treatment effect from indirect evidence; RoR = ratio of ratios; Z = z-value for disagreement between direct and indirect evidence; P-value = p-value for inconsistency.

**Supplementary Table 6.** Egger’s regression test

| Outcomes | P-value (2-tailed) |
| --- | --- |
| Major Adverse Cardiovascular Events | 0.97 |
| Major bleeding (BARC 3–5) | 0.16 |
| TIMI major bleeding | 0.79 |
| PLATO major bleeding | 0.89 |
| All-cause death | 0.66 |
| Cardiovascular death | 0.80 |
| Myocardial infarction | 0.72 |
| Stroke | 0.70 |
| Stent thrombosis | 0.47 |
| Dyspnea | 0.11 |

**Supplementary Table 7.** Summary of the Studies Included in This Review

| **Study ID** | **Study design** | **Country** | **Time frame (recruitment period)** | **Follow-up duration** | **Total sample size** | **Population** | **Groups** | | **Inclusion criteria** | **Primary outcome** | **Conclusion** |
| --- | --- | --- | --- | --- | --- | --- | --- | --- | --- | --- | --- |
|  |  |  |  |  |  |  | **Intervention** | **Comparator** |  |  |  |
| Angiolillo, 2011 (OPTIMUS-3 Trial) | Prospective, randomized, double-blind, active-controlled, two-period crossover study | USA | April 2008 to January 2009 | Two 1-week treatment periods separated by a 2-week washout | 35 | Patients with type 2 DM and CAD taking aspirin | Prasugrel 60 mg LD → 10 mg MD plus aspirin | Clopidogrel 600 mg LD → 150 mg MD plus aspirin | Participants were aged 18–75 years with type 2 DM treated for at least 1 month with oral and/or parenteral hypoglycemic therapy, with known CAD, and receiving aspirin 81–325 mg/day. | Inhibition of platelet aggregation by VerifyNow P2Y12 assay at 4 h after loading dose | Standard-dose prasugrel produced greater platelet inhibition and better response profiles than double-dose clopidogrel in aspirin-treated patients with type 2 DM and CAD. |
| Alexopoulos, 2013 | Prospective, randomized, single-center, single-blind, crossover study | Greece | June 2012 to September 2012 | Two 15-day treatment periods without washout | 30 | Type 2 DM patients with ACS undergoing PCI and pretreated with clopidogrel | Ticagrelor 180 mg LD → 90 mg BID MD plus aspirin | Prasugrel 60 mg LD → 10 mg MD plus aspirin | Patients with type 2 DM presenting with ACS undergoing PCI with DES implantation, who were pretreated with clopidogrel | PR at the end of the two study periods assessed by VerifyNow P2Y12 assay and measured in PRU | Ticagrelor achieved significantly higher platelet inhibition than prasugrel; both agents effectively treated HPR. |
| Mangiacapra, 2016 (CLOTILDIA) | Prospective, randomized, single-center, open-label, crossover study | Italy | NR | 28 days | 42 | Type 2 DM patients with stable CAD treated with PCI and DES on DAPT | Ticagrelor 90 mg BID plus aspirin | Clopidogrel 150 mg once daily plus aspirin | Type 2 DM patients with stable CAD undergoing PCI with DES, on aspirin + clopidogrel, at least 1-month post-PCI. | Endothelial function assessed by FMD and NMD; platelet reactivity assessed by PRU (VerifyNow) | Ticagrelor resulted in lower platelet reactivity and improved endothelial function compared with high dose clopidogrel in DM patients with stable CAD. |
| Franchi, 2016 (OPTIMUS-4) | Prospective, randomized, double-blind, crossover pharmacodynamic study | USA | February 2013 to July 2015 | 1 week treatment + 2- to 4-week washout + 1 week crossover treatment | 50 | Aspirin-treated patients with type 2 DM and CAD | Ticagrelor 180 mg LD → 90 mg BID MD plus aspirin | Prasugrel 60 mg LD → 10 mg MD plus aspirin | Patients aged 18–74 years with type 2 DM on oral hypoglycemic agents or insulin, with angiographically documented CAD (> 50% stenosis in a major epicardial coronary vessel), and on aspirin 81 mg/day for at least 30 days. | PRU by VerifyNow P2Y12 at 1 week between prasugrel and ticagrelor | In patients with DM and CAD, ticagrelor exerted similar or greater inhibition of ADP-induced platelet reactivity than prasugrel, while non-ADP-induced platelet reactivity did not differ significantly between treatments. |
| He, 2021 | Single-center, prospective, randomized, open-label, controlled registry trial | China | October 2017 to March 2019 | 6 months | 270 | Patients with ACS and type 2 DM undergoing PCI | Ticagrelor 180 mg LD → 90 mg BID MD plus aspirin | Clopidogrel 300–600 mg LD → 75 mg MD plus aspirin | Patients with ACS (defined as UAP or acute MI) who underwent PCI, with type 2 DM defined as fasting blood glucose ≥ 126 mg/dL, random blood glucose ≥ 200 mg/dL, or known DM on hypoglycemic therapy. | Composite of nonfatal MI, target vessel revascularization, rehospitalization, stroke, and death from any cause at 6 months | Ticagrelor did not improve the composite efficacy outcome versus clopidogrel but significantly increased BARC-defined bleeding during 6-month follow-up in Chinese patients with ACS and DM. |
| Bangalore, 2026 (TUXEDO-2) | Prospective, multicenter, open-label, randomized clinical trial | India | February 2020 to August 2024 | 1 year | 1,800 | Patients with DM and multivessel CAD undergoing PCI | Ticagrelor 180 mg LD → 90 mg BID MD plus aspirin | Prasugrel 60 mg LD → 10 mg MD plus aspirin | Patients aged ≥ 18 years with DM, multivessel CAD, undergoing PCI. | Composite of death, nonfatal MI, stroke, or BARC major bleeding at 1 year | Ticagrelor was not noninferior to prasugrel for the primary composite outcome in patients with DM and multivessel CAD undergoing PCI. |
| James, 2010 (PLATO DM substudy) | Prespecified subgroup analysis of a randomized clinical trial | Multinational | NR | Median study treatment 9.1 months | 4,662 | Patients with ACS and pre-existing DM from the PLATO trial | Ticagrelor 180 mg LD → 90 mg BID MD plus aspirin | Clopidogrel 300–600 mg LD → 75 mg MD plus aspirin | Patients from PLATO with STEMI or NSTEMI ACS within the previous 24 h, with a DM subgroup defined by investigator-reported pre-existing DM. | Composite of CV death, MI, or stroke; primary safety was PLATO-defined major bleeding | Ticagrelor reduced ischemic events in ACS patients irrespective of DM status and glycemic control, without an increase in major bleeding events. |
| Laine, 2014 | Single-center, prospective, open-label, randomized study | France | October 2012 to February 2013 | In-hospital; platelet reactivity assessed 6 to 18 h post-LD | 100 | Patients with DM undergoing PCI for ACS | Ticagrelor 180 mg LD → 90 mg BID MD ± aspirin | Prasugrel 60 mg LD → 10 mg MD ± aspirin | Patients with DM on stable chronic medical therapy for at least 3 months, presenting with ACS, selected for an invasive strategy, and undergoing PCI. | PR following LD of P2Y12 receptor antagonist measured by VASP index | Ticagrelor LD was superior to prasugrel LD in reducing PR in ACS patients with DM. |
| Li, 2019 | Single-center, prospective, randomized controlled study | China | January 2011 to January 2015 | 6 months | 200 | Patients with STEMI and type 2 DM undergoing PCI | Ticagrelor 180 mg LD → 90 mg BID MD | Clopidogrel 300 mg LD → 75 mg MD | Adult patients with STEMI diagnosed with ESC guidelines, DM defined according to ADA criteria, and all patients undergoing PCI. | MACE including MI, angina, HF, stent thrombosis, and mortality; cardiac biomarkers and function | Ticagrelor improved myocardial function and reduced MACE compared with clopidogrel in STEMI patients with DM. |
| Liu, 2020 (PLATIDE-PCI) | Single-center, prospective, randomized, open-label, controlled study | China | May 2016 to December 2018 | 15 days | 40 | Patients with type 2 DM after recent PCI for CCS | Ticagrelor 180 mg LD → 90 mg BID MD plus aspirin | Clopidogrel 300–600 mg LD → 75 mg MD plus aspirin | Patients aged ≥18 years with documented CCS, type 2 DM, who were at least 24 h but within 14 days after a recent successful elective PCI, and receiving steady-state DAPT with aspirin and clopidogrel. | PRU 2 to 4 h after final study dose on day 15 | Ticagrelor achieved greater peak and trough platelet inhibition than clopidogrel in diabetic patients after recent PCI for CCS. |
| Marcano, 2022 (TICS-DM) | Prospective, open-label, randomized, crossover pharmacodynamic study | Spain | NR | 1 week treatment per regimen with 2–4-week washout between regimens | 25 | Mediterranean patients with type 2 DM and CCS on aspirin therapy | Ticagrelor 180 mg LD, then 90 mg BID plus aspirin | Clopidogrel 600 mg LD, then 75 mg daily plus aspirin | Patients aged 18–75 years with type 2 DM, stable angiographically documented CAD/CCS, and receiving aspirin therapy. | MPA measured by LTA with 20 µM ADP after 1 week of therapy | In Mediterranean patients with DM and CCS, ticagrelor provided more potent antiplatelet effect than clopidogrel after LD and during maintenance therapy. |
| Ndrepepa, 2020 (ISAR-REACT 5 DM analysis) | Pre-specified subgroup analysis of a randomized clinical trial | Multicenter, Europe | September 2013 to October 2018 | 12 months | 892 | Patients with ACS and DM in whom invasive treatment was planned | Ticagrelor 180 mg LD, then 90 mg BID plus aspirin | Prasugrel 60 mg LD, then 10 mg daily plus aspirin | Patients with ACS (including USA, NSTEMI, or STEMI) with planned invasive therapy, and DM defined by insulin or oral hypoglycemic treatment on admission | Composite of death, MI, or stroke at 12 months | In patients with ACS and DM, the efficacy of ticagrelor was comparable with that of prasugrel. |
| Bhatt, 2016 (PEGASUS–TIMI 54) | Randomized controlled trial (pre-specified subgroup analysis) | Multicenter (international) | October 2010 to May 2013 | 33 months (median) | 6,806 | Patients with prior MI (1–3 years) and DM | Ticagrelor 90 mg BID plus aspirin | placebo plus aspirin | Patients with prior MI 1–3 years earlier and ≥1 additional risk factor, with the DM subgroup analyzed separately. | MACE (CV death, MI, stroke); primary safety endpoint TIMI major bleeding | Ticagrelor 90 mg BID reduced ischemic events numerically versus placebo in DM patients, with increased TIMI major bleeding. |
| Steg, 2019 (THEMIS) | Randomized, double-blind, placebo-controlled trial | Multinational | February 10, 2014, to May 24, 2016 | Median 39.9 months | 19,220 | Patients with stable CAD and type 2 DM without prior MI or stroke | Ticagrelor 90 mg BID MD plus aspirin | Placebo plus aspirin | Patients aged ≥50 years with stable CAD defined by prior PCI or CABG or angiographic stenosis ≥50% in at least 1 coronary artery, type 2 DM treated with antihyperglycemic medication for at least 6 months, and no prior MI or stroke. | Composite of CV death, MI, or stroke | In patients with stable CAD and DM without prior MI or stroke, ticagrelor plus aspirin reduced ischemic CV events but increased major bleeding versus placebo plus aspirin. |
| Wiviott, 2008 (TRITON–TIMI 38 DM subgroup) | Prespecified subgroup analysis of a randomized clinical trial | Multinational | November 2004 to January 2007 | 450 days | 3,146 | Patients with ACS and preexisting DM undergoing PCI | Prasugrel 60 mg LD → 10 mg MD plus aspirin | Clopidogrel 300 mg LD → 75 mg MD plus aspirin | Patients with moderate- to high-risk UA/NSTEMI, STEMI after medical treatment with coronary anatomy suitable for PCI, or STEMI with planned primary PCI, and preexisting DM. | Composite of CV death, nonfatal MI, or nonfatal stroke | Prasugrel reduced ischemic events without an observed increase in TIMI major bleeding and provided greater net clinical benefit than clopidogrel. |
| Jeong, 2017 | Prospective, randomized, open-label, crossover trial | Republic of Korea | July 2015 to April 2016 | 10 weeks | 62 | Type 2 DM patients with NSTE-ACS requiring coronary stenting | Ticagrelor 180 mg LD, then 90 mg BID plus aspirin | Prasugrel 60 mg LD, then 10 mg daily plus aspirin | Patients aged 35–74 years with newly diagnosed type 2 DM or type 2 DM on hypoglycemic agents, presenting with NSTE-ACS with successful coronary stent implantation and TIMI flow grade 3 after the procedure. | Comparison of changes in vascular function | Ticagrelor significantly reduced inflammatory cytokines and increased circulating EPCs compared to prasugrel. |
| Dalby, 2017 (TRILOGY ACS DM analysis) | Prespecified subgroup analysis of a randomized, double-blind, double-dummy, active-control trial | Multinational | NR | Up to 30 months | 3,539 | NSTE-ACS patients with DM managed medically without revascularization | Prasugrel 10 mg once daily MD plus aspirin | Clopidogrel 75 mg once daily MD plus aspirin | Patients with NSTE-ACS managed without revascularization, and DM defined as treatment with diet, oral hypoglycemic agents, and/or insulin. | Composite of CV death, MI, or stroke | Patients with DM had higher ischemic risk, especially with insulin treatment, and there was no differential treatment effect of prasugrel vs. clopidogrel |

ACS: acute coronary syndrome; NSTE-ACS: non–ST-segment elevation acute coronary syndrome; STEMI: ST-segment elevation myocardial infarction; NSTEMI: non–ST-segment elevation myocardial infarction; UAP: unstable angina pectoris; CCS: chronic coronary syndrome; CAD: coronary artery disease; DM: diabetes mellitus; PCI: percutaneous coronary intervention; CABG: coronary artery bypass grafting; DES: drug-eluting stent; DAPT: dual antiplatelet therapy; LD: loading dose; MD: maintenance dose; BID: twice daily; MACE: major adverse cardiovascular event; MI: myocardial infarction; CV: cardiovascular; HF: heart failure; PR: platelet reactivity; PRU: P2Y12 reaction units; HPR: high platelet reactivity; LTA: light transmission aggregometry; ADP: adenosine diphosphate; VASP: vasodilator-stimulated phosphoprotein; FMD: flow-mediated dilation, NMD: nitroglycerin-mediated dilation; BARC: Bleeding Academic Research Consortium; TIMI: Thrombolysis in Myocardial Infarction; ESC: European Society of Cardiology; ADA: American Diabetes Association; EPCs: endothelial progenitor cells.

**Supplementary Table 8.** Baseline Demographic Characteristics of the Study Population

| **Study ID** | **Groups (n)** | **Demographics** | | | | **Clinical presentation** | | | | **Medical history, n (%)** | | | | | | | **Laboratory variables, mean (SD)** | |
| --- | --- | --- | --- | --- | --- | --- | --- | --- | --- | --- | --- | --- | --- | --- | --- | --- | --- | --- |
|  |  | **Age, mean (SD)** | **BMI, mean (SD)** | **Sex, n (%)** | | **ACS** | | | **CCS** | **Current smoking** | **HTN** | **Dyslipidemia** | **Prior MI** | **Prior PCI** | **Prior CABG** | **Prior stroke/TIA** | **HbA1c, %** | **Creatinine, mg/dL** |
|  |  |  |  | **Male** | **Female** | **STEMI** | **NSTEMI** | **USA** |  |  |  |  |  |  |  |  |  |  |
| Angiolillo, 2011 (OPTIMUS-3 Trial) | Total randomized = 35 | 61.3 (8.8) | 33.1 (8.2) | 24 (68.6) | 11 (31.4) | NR | NR | NR | 35 (100) | 7 (20.0) | 33 (94.3) | 33 (94.3) | 13 (37.1) | 20 (57.1) | 8 (22.9) | NR | NR | NR |
| Alexopoulos, 2013 | Ticagrelor (n = 15) | 65.4 (7.7) | 29.5 (3.9) | 14 (93.3) | 1 (6.7) | 3 (20.0) | 7 (46.7) | 5 (33.3) | 0 (0) | 6 (40.0) | 10 (66.7) | 8 (53.3) | 4 (26.7) | 3 (20.0) | 1 (6.7) | 0 (0) | 7.4 (0.7) | NR |
|  | Prasugrel (n = 15) | 60.9 (8.0) | 28.9 (4.6) | 14 (93.3) | 1 (6.7) | 4 (26.7) | 4 (26.7) | 7 (46.7) | 0 (0) | 5 (33.3) | 11 (73.3) | 7 (46.7) | 3 (20.0) | 4 (26.7) | 0 (0) | 0 (0) | 7.7 (1.0) | NR |
| Mangiacapra, 2016 (CLOTILDIA) | Overall cohort (N = 42) | NR | NR | NR | NR | NR | NR | NR | NR | NR | NR | NR | NR | NR | NR | NR | NR | NR |
| Franchi, 2016 (OPTIMUS-4) | Overall PD population (N = 46) | 59 (8) | 36 (7) | 33 (72) | 13 (28) | NR | NR | NR | 46 (100) | 11 (24) | 43 (93) | 40 (87) | 25 (54) | 31 (67) | 17 (37) | NR | 7.5 (1.2) | 1.0 (0.4) |
| He, 2021 | Ticagrelor (n = 135) | 62.7 (8.9) | 25.1 (3.4) | 39 (29.3) | 94 (70.7) | 19 (14.3) | 18 (13.5) | 96 (72.2) | 0 (0) | 73 (54.9) | 80 (60.2) | 28 (21.1) | 21 (15.8) | 26 (19.5) | NR | 9 (6.8) | 7.9 (1.8) | 0.87 (0.20) |
|  | Clopidogrel (n = 135) | 63.3 (8.9) | 24.8 (3.0) | 47 (35.3) | 86 (64.7) | 13 (9.8) | 17 (12.8) | 103 (77.4) | 0 (0) | 68 (51.1) | 96 (72.2) | 29 (21.8) | 12 (9.8) | 20 (15.0) | NR | 13 (9.8) | 7.7 (1.4) | 0.85 (0.23) |
| Bangalore, 2026 (TUXEDO-2) | Ticagrelor (n = 901) | 60.48 (10.19) | 24.69 (3.90) | 636 (70.6) | 265 (29.4) | 220 (24.4) | 308 (34.2) | 189 (21.0) | 184 (20.4) | 140 (15.5) | 534 (59.3) | 749 (83.1) | 183 (20.3) | 31 (3.4) | 3 (0.3) | 19 (2.1) | 7.99 (1.79) | 1.07 (0.73) |
|  | Prasugrel (n = 899) | 60.09 (10.17) | 24.80 (3.86) | 660 (73.4) | 239 (26.6) | 233 (25.8) | 299 (33.3) | 172 (19.1) | 196 (21.8) | 146 (16.2) | 550 (61.2) | 748 (83.2) | 204 (22.7) | 43 (4.8) | 5 (0.6) | 19 (2.1) | 8.08 (1.88) | 103 (0.39) |
| James, 2010 (PLATO DM substudy) | Ticagrelor (n = 2,326) | 64 (11.8) | 28.96 (4.97) | 1,537 (66.1) | 789 (33.9) | 655 (28.2) | 1,125 (48.4) | 482 (20.7) | 0 (0) | 573 (24.6) | 1,902 (81.8) | 1,361 (58.5) | 629 (27.0) | 428 (18.4) | 242 (10.4) | 198 (8.5) | 7.73 (1.63) | NR |
|  | Clopidogrel (n = 2,336) | 64 (11.8) | 28.6 (4.5) | 1,501 (64.3) | 835 (35.7) | 681 (29.2) | 1,092 (46.7) | 493 (21.1) | 0 (0) | 583 (25.0) | 1,900 (81.3) | 1,421 (60.8) | 632 (27.1) | 419 (17.9) | 222 (9.5) | 228 (9.8) | 7.77 (1.85) | NR |
| Laine, 2014 | Ticagrelor group (n = 50) | 64.8 (8.9) | 27.6 (3.1) | 33 (66) | 17 (34) | NR | NR | 9 (18.8) | 0 (0) | 14 (28) | 40 (80) | 28 (56) | NR | 15 (30) | 5 (10) | 0 (0) | 7.5 (1.2) | 0.95 (0.40) |
|  | Prasugrel group (n = 50) | 62.8 (8.2) | 28.5 (5.5) | 43 (86) | 7 (14) | NR | NR | 10 (20) | 0 (0) | 14 (28) | 35 (70) | 31 (62) | NR | 11 (22) | 2 (4) | 0 (0) | 7.3 (1.3) | 0.95 (0.30) |
| Li, 2019 | Ticagrelor (n = 100) | 70.8 (8.9) | NR | 55 (55) | 45 (45) | 100 (100) | 0 (0) | 0 (0) | 0 (0) | 36 (53.1) | 29 (38.8) | 27 (34.7) | NR | NR | NR | NR | NR | NR |
|  | Clopidogrel (n = 100) | 79.1 (9.4) | NR | 61 (61) | 39 (39) | 100 (100) | 0 (0) | 0 (0) | 0 (0) | 38 (57.1) | 26 (38.8) | 29 (38.8) | NR | NR | NR | NR | NR | NR |
| Liu, 2020 (PLATIDE-PCI) | Ticagrelor (n = 20) | 62.9 (7.6) | 26.5 (2.8) | 12 (60.0) | 8 (40.0) | 0 (0) | 0 (0) | 0 (0) | 20 (100.0) | 8 (40.0) | 15 (75.0) | 12 (60.0) | 1 (5.0) | 5 (25.0) | 0 (0.0) | NR | 7.5 (1.3) | NR |
|  | Clopidogrel (n = 19) | 63.3 (10.4) | 27.4 (3.3) | 15 (78.9) | 4 (21.1) | 0 (0) | 0 (0) | 0 (0) | 19 (100.0) | 7 (36.8) | 14 (73.7) | 13 (68.4) | 2 (10.5) | 2 (10.5) | 0 (0.0) | NR | 7.2 (1.4) | NR |
| Marcano, 2022 (TICS-DM) | Overall cohort (N = 20) | 65.45 (4.88) | 29.6 (3.8) | 16 (80) | 4 (20) | 0 (0) | 0 (0) | 0 (0) | 20 (100) | 1 (5) | 16 (80) | 18 (90) | 14 (70) | 17 (85) | 4 (20) | 0 (0) | 7.0 (1.1) | NR |
| Ndrepepa, 2020 (ISAR-REACT 5) | Ticagrelor (n = 463) | 66.8 (11.0) | 29.5 (5.1) | 342 (73.9) | 121 (26.1) | 166 (35.9) | 233 (50.3) | 64 (13.8) | 0 (0) | 117 (25.5) | 399 (86.4) | 318 (69.0) | 102 (22.1) | 152 (32.9) | 35 (7.6) | NR | NR | 0.93 (0.35) |
|  | Prasugrel (n = 429) | 67.5 (11.5) | 29.2 (4.6) | 332 (77.4) | 97 (22.6) | 146 (34.1) | 216 (50.3) | 67 (15.6) | 0 (0) | 95 (22.4) | 361 (84.3) | 298 (69.5) | 81 (18.9) | 140 (32.7) | 51 (11.9) | NR | NR | 0.94 (0.35) |
| Bhatt, 2016 (PEGASUS–TIMI 54) | Ticagrelor (n = 4,549) | 63.0 (NR) | 29.1 (NR) | 3,303 (72.6) | 1,246 (27.4) | 2,360 (52.0) | 1,926 (42.4) | NR | 4,549 (100) | 716 (15.7) | 3,889 (85.5) | 3,605 (79.2) | 4,549 (100) | 3,627 (79.7) | 237 (5.2) | 92 (2.0) | NR | NR |
|  | Placebo (n = 2,257) | 64.0 (NR) | 29.3 (NR) | 1,615 (71.6) | 642 (28.4) | 1,182 (52.5) | 932 (41.4) | NR | 2,257 (100) | 345 (15.3) | 1,930 (85.5) | 1,789 (79.3) | 2,257 (100) | 1,792 (79.4) | 134 (5.9) | 37 (1.6) | NR | NR |
| Steg, 2019 (THEMIS) | Ticagrelor (n = 9,619) | 66.3 (8.1) | 29.2 (4.8) | 6,576 (68.4) | 3,043 (31.6) | 0 (0) | 0 (0) | 0 (0) | 9,619 (100) | 1,056 (11.0) | 8,909 (92.6) | 8,386 (87.2) | 0 (0) | 5,558 (57.8) | 2,120 (22) | 0 (0) | 7.2 (1.3) | NR |
|  | Placebo (n = 9,601) | 66.3 (8.1) | 29.3 (5.0) | 6,613 (68.9) | 2,988 (31.1) | 0 (0) | 0 (0) | 0 (0) | 9,601 (100) | 1,038 (10.8) | 8,867 (92.4) | 8,367 (87.1) | 0 (0) | 5,596 (58.3) | 2,071 (21.6) | 0 (0) | 7.2 (1.3) | NR |
| Wiviott, 2008 (TRITON–TIMI 38) | Overall DM cohort (n = 3,146) | 63 (11.9) | 29 (5.2) | 2,108 (67) | 1,038 (33) | 661 (21) | 2,485 (79) | NR | 0 (0) | 849 (27) | 2,517 (80) | 2,108 (67) | 724 (23) | NR | 378 (12) | 189 (6) | NR | NR |
| Jeong, 2017 | Ticagrelor (n = 31) | 62.0 (9.2) | 26.1 (3.5) | 24 (77.4) | 7 (22.6) | 0 (0) | NR | NR | 0 (0) | 16 (51.6) | 21 (67.7) | NR | NR | 0 (0) | 0 (0) | 0 (0) | NR | 0.98 (0.19) |
|  | Prasugrel (n = 31) | 60.2 (9.2) | 25.8 (2.9) | 21 (67.7) | 10 (32.3) | 0 (0) | NR | NR | 0 (0) | 13 (41.9) | 23 (74.2) | NR | NR | 0 (0) | 0 (0) | 0 (0) | NR | 0.91 (0.19) |
| Dalby, 2017 (TRILOGY ACS) | Overall DM cohort (n = 3,539) | 64 (11.9) | NR | 1,999 (56.5) | 1,540 (43.5) | 0 (0) | 2,513 (71.0) | 1,026 (29.0) | 0 (0) | 605 (17.1) | 3,089 (87.3) | 2,272 (64.2) | 1,465 (41.4) | 941 (26.6) | 676 (19.1) | NR | NR | 1.0 (0.3) |
